# Fine scale hippocampus morphology variation cross 552 healthy subjects from age 20 to 80

**DOI:** 10.3389/fnins.2023.1162096

**Published:** 2023-08-31

**Authors:** Qinzhu Yang, Shuxiu Cai, Guojing Chen, Xiaxia Yu, Renee F. Cattell, Tammy Riklin Raviv, Chuan Huang, Nu Zhang, Yi Gao

**Affiliations:** ^1^School of Biomedical Engineering, Shenzhen University Medical School, Shenzhen University, Shenzhen, Guangdong, China; ^2^Department of Biomedical Engineering, Stony Brook University, Stony Brook, NY, United States; ^3^Department of Radiation Oncology, Renaissance School of Medicine, Stony Brook University, Stony Brook, NY, United States; ^4^The School of Electrical and Computer Engineering, Ben-Gurion University of the Negev, Be'er Sheva, Israel; ^5^Department of Psychiatry, Stony Brook University, Stony Brook, NY, United States; ^6^Department of Radiology, Stony Brook University, Stony Brook, NY, United States; ^7^Department of Neurosurgery, The First Affiliated Hospital of Sun Yat-sen University, Guangzhou, Guangdong, China; ^8^Shenzhen Key Laboratory of Precision Medicine for Hematological Malignancies, Shenzhen, China; ^9^Marshall Laboratory of Biomedical Engineering, Shenzhen University, Shenzhen, China

**Keywords:** hippocampus, fine-scale segmentation, shape analysis, deep learning, MRI

## Abstract

The cerebral cortex varies over the course of a person's life span: at birth, the surface is smooth, before becoming more bumpy (deeper sulci and thicker gyri) in middle age, and thinner in senior years. In this work, a similar phenomenon was observed on the hippocampus. It was previously believed the fine-scale morphology of the hippocampus could only be extracted only with high field scanners (7T, 9.4T); however, recent studies show that regular 3T MR scanners can be sufficient for this purpose. This finding opens the door for the study of fine hippocampal morphometry for a large amount of clinical data. In particular, a characteristic bumpy and subtle feature on the inferior aspect of the hippocampus, which we refer to as hippocampal dentation, presents a dramatic degree of variability between individuals from very smooth to highly dentated. In this report, we propose a combined method joining deep learning and sub-pixel level set evolution to efficiently obtain fine-scale hippocampal segmentation on 552 healthy subjects. Through non-linear dentation extraction and fitting, we reveal that the bumpiness of the inferior surface of the human hippocampus has a clear temporal trend. It is bumpiest between 40 and 50 years old. This observation should be aligned with neurodevelopmental and aging stages.

## 1. Introduction

Numerous radiological studies of sub-cortical morphology have shown many brain disorders to be correlated with hippocampal shape (Styner et al., 2004; Thompson et al., 2004; Apostolova et al., 2006; Wang et al., 2006; Scher et al., 2007; Colliot et al., 2008; Nestor et al., 2013; Gao et al., 2014; Gao and Bouix, 2016), volume (Fleisher et al., 2008), or metabolic properties (Kraguljac et al., 2013). The hippocampus also exhibits important related variations in healthy individuals. For example, spatial memory declines with age and this is consistent with a decreasing trend in hippocampal volume (Bohbot et al., 2004; Konishi et al., 2017). Moreover, the hippocampal structure also correlates with the function of establishing semantic associations in memory (Henke et al., 1999). As people age, the rate of hippocampal atrophy increases, with the greatest increase after middle age (Fraser et al., 2015). These comparable global features of non-clinical and clinical conditions (Convit et al., 1997; Schuff et al., 2009) provide an important measurement for the evaluation of hippocampal abnormalities and functions.

Morphological and functional assessment of fine-scale structures are still considered challenging tasks. The hippocampus is known to be among the few structures where neurogenesis continues to take place after birth. Similar to the formation of any other cortical gyrus/sulcus, the proliferation and stacking of cells in hippocampal neuronal layers requires space-efficient outward folding of the hippocampal surface. Furthermore, it is worth noting that hippocampus neurogenesis-associated features exhibit both qualitative and quantitative age-related alterations (Knoth et al., 2010). This work aims to investigate the macroscopic morphological appearance and its age-dependent variability across the life span of the hippocampus.

**Figure 1:**
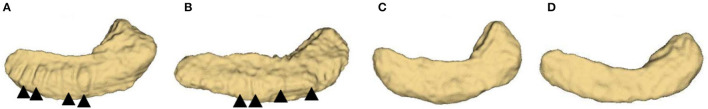
Inferior view of four hippocampus. **(A, B)**: bumpy group; **(C, D)**: smooth group. The arrowheads indicate the prominent dentations and their approximate orientations.

The structure of hippocampal dentation is of particular interest due to its apparent rugged ridges, which are in the CA1/subiculum on the inferior aspect of the hippocampal body and extend through the inferior medial aspect of the tail (Duvernoy, 2013). Consulting neuroanatomy textbook (Duvernoy et al., 2005; Arslan, 2014; Ribas, 2018; ten Donkelaar et al., 2018), the dentated appearance is obvious and exhibits great variability of shape as shown in Figure 1. Unfortunately, this variability has been largely overlooked in previous image based studies.

Such morphological variation mostly involves the CA1 regions. CA1 neurons are known to be involved in episodic memory (Bartsch et al., 2011) and a positive correlation between cortical gyrification and cognitive functioning was found (Luders et al., 2008). Further quantitative studies related to episodic memory (Beattie et al., 2017) have used ultra-high resolution MRI data to explore the highly variable long axis of hippocampal dentation and its functional role in episodic memory.

Quantitative feature generation would be a valuable tool for the intuitive, concise, and personalized characterization of hippocampal dentation. Moreover, hippocampal dentation varies across individuals, over time and along the inferior surface. This variation makes it significant for quantifying the relationship between hippocampal dentation and other factors, such as age, clinical, or non-clinical conditions. More importantly, the quantitative analysis of fine-scale structures allows us to leverage advanced machine learning methods and enables us to explore data sets more extensively.

There are two main reasons why current research is insufficient to quantify hippocampal dentation changes. First, quantitative research methods usually require a large data size, but the limited acquisition of high resolution image data with hippocampal fine-scale structure leads to difficulties in large-scale research. The main reason for this is because clinical 3T scanners find it difficult to acquire sufficient resolution and the currently finite availability of ultra-high field scanners (7T or greater) (Wisse et al., 2012; Kim et al., 2013; Derix et al., 2014) or post-mortem specimens (Yushkevich et al., 2009). Second, compared to global structure, fine-scale structure is difficult to characterize by most handcrafted feature representation in feature engineering (Bengio et al., 2013) or automatic extraction of features through deep learning networks. This may be due to the small, hard-to-measure structural geometry and the challenge of properly delineating regional boundaries. Additional challenges stem from dentate variability along the different sagittal slices of hippocampal dentation.

The above aspects have made it difficult to conduct a quantitative analysis of the dentated shape of the hippocampus. The most closely related work by Kilpattu Ramaniharan et al. (2022) visualized dentation after using the up-sampling method and ASHS software. They counted dentation and explored its association with memory dysfunction in patients with temporal lobe epilepsy that have hippocampal sclerosis. Beattie et al. (2017) visualized the dentation using ultra-high resolution structural MRI and using a visual rating scale, accessed by human observers, which showed that the extent of dentation varied considerably across individuals and was positively correlated with memory recall and visual memory recognition. The raters in that study needed to examine all sagittal slices to observe dentation visible through the entire width of the hippocampus. This work is labor-intensive, highly subjective, and can suffer from high intra- and inter- reader variability. Therefore, this rating scheme cannot be generalized reliably to a large number of subjects across multiple institutions. A computed aided quantification and analysis framework for evaluating the hippocampal dentation is therefore needed to provide objective fine-scale morphometry.

This analysis framework consists of two components. First, an effective and efficient segmentation algorithm is needed that is capable of capturing fine-scale dentations. It has been shown previously that such local and subtle features under the hippocampus can be reconstructed from clinical 3T MRI by a multi-atlas based technique (Chang et al., 2018). However, the multi-atlas warping technique (Nestor et al., 2013) could not fulfill the further need for a large population study due to it being extremely time-consuming. More recently, the use of a 3D deep convolutional neural network for hippocampus segmentation has achieved high precision, measured by a global metric such as the Dice coefficient (Thyreau et al., 2018). Part of its training labels was from the FreeSurfer algorithm (Fischl, 2012). Using the synthetic data and augmentation algorithm, the Dice average coefficients are above 90%. Later, in a hippocampal segmentation study of a stroke population, Zavaliangos-Petropulu et al. (2022) used deep learning based Hippodeep method (Thyreau et al., 2018) and make a comparison with FreeSurfer. Rather than achieving annotation with the help of Freesurfer, Goubran et al. (2020) trained the CNN using 259 bilateral manually delineated segmentations to achieve better performance. Guo et al. (2020) proposed a longitudinal classification-regression model for segmenting the hippocampus in infant brain MRIs. Work by Liu et al. (2020) proposed a joint automatic hippocampal segmentation and AD classification method. For refined segmentation by exploiting space information, Pang et al. (2019) proposed a method based on iterative local linear mapping (ILLM) with representative and local structure-preserved feature embedding. To improve segmentation quality, Van Opbroek et al. (2018) and Ataloglou et al. (2019) explored different transfer learning techniques.

Even though current CNN based hippocampus segmentation methods have achieved global accuracy measures, they still lack the ability of the 3T images to capture fine-scale dentations. Moreover, to conduct large scale statistical morphological studies, the generalization capability of the CNN needs to be strengthened to handle the image intensity fluctuations among different scans, machine-dependent noise, and bias field in-homogeneity, etc. To address such issues, Memmel utilized the data from different domains with the GAN framework to disregard domain-specific information (Memmel et al., 2021). For hippocampus segmentation across different datasets, few studies have considered how to solve this with the help of domain adaptation in an end-to-end framework directly. In response, our proposed framework can be adopted in these similar studies. To further improve the framework, some research by Strudel et al. (2021) and Valanarasu et al. (2021) initially used the transformer block to extract features and process the long range relationship of these features. To utilize these techniques we need to improve the feature extraction ability of the design framework before subsequent discriminator and segmentation steps, noting that based segmentation operation is the foundation to help for the subsequent fine-scale segmentation and accurate shape analysis.

Once the hippocampus is successfully segmented and its fine-scale morphology features are extracted, we need to design a technique that specifically compares the dentated structures underneath the CA1 region. Previously, the analysis of shapes is usually conducted between two groups of shapes, trying to identify the region where the two groups of shapes differ significantly (Gerig et al., 2001; Shen and Makedon, 2006; Styner et al., 2006; Cates et al., 2008; Shen et al., 2009; Shen, 2010; Riklin Raviv et al., 2014; Hong et al., 2015; Gao and Bouix, 2016). However, the scenario is different in this work as we have already identified certain regions on the shape, as well as the possible pattern of variation. We are more explicitly interested in the magnitude of the dentated pattern between the two groups. To this end, we have to design a suitable approach to handle the problem at hand.

As an exploratory proposition, we hypothesize that the level of dentations may be involved in neurogenesis with age, reflected by variation of dentated structure along its long axis. This work presents a novel domain adaption segmentation and regression model of quantitative features on a relatively large dataset of 552 subjects (1,104 hippocampi) (IXI dataset, 2018). As a key step in the successful application of machine learning for quantitative estimation (Bengio et al., 2013), to handle the great variability of hippocampal dentation, we combined the advantages of domain adaption segmentation in the field of deep learning and propose a new feature representation method for dentation analysis.

Using deep learning methods, we have designed a transformer based approach to segment and extract the hippocampus. This approach is then combined with the learned grayscale information of the hippocampus, and multi-scale segmentation is performed to obtain fine-scale segmentation. Once the dentated structures are extracted, we then measure the magnitude of the dentation structure by first identifying the long axis of the hippocampus. This is done under a point cloud representation of the shape. Then, specifically engineered for the dentation under the CA1 region, a non-linear fitting of the sinusoidal function is performed; the observation that the dentation presents an arciform or sinusoidal appearance allows us to quantify the convolution by magnitude and frequency of the sinusoidal function. Moreover, using simulated annealing, we can find the most optimal model parameters.

Our work contributes to the field in three ways: (1) A deep-learning based robust segmentation algorithm is used to extract the fine-scale hippocampal morphological feature at the sub-pixel level on a large dataset. (2) This study demonstrates that certain fine-scale hippocampal morphological features vary with aging. (3) To our knowledge, even though rich hippocampal shape studies have been conducted previously, this is the first fine-scale quantitative analysis on hippocampal dentation based on a clinically available dataset. Our method aims to study the differences in hippocampal shape of a healthy population over an age range from people aged in their mid-20s to 80 years old. The construction of an analytical baseline and the development of a technique for robust and quantitative image analysis will open possibilities for future comparisons between non-clinical and clinical groups.

## 2. Materials and methods

To use the hippocampus segmentation algorithm, 41 3T MR images with the hippocampus manually traced out were used to train the segmentation model. These 41 cases are from the EADC-ADNI Harmonized Protocol project (Apostolova et al., 2015; Boccardi et al., 2015; Frisoni et al., 2015). We also performed segmentation validation with real 7T MR images based on samples from Alkemade et al. (2020). For aging hippocampus morphology research, the hippocampi of 552 healthy subjects (age range: 20–79, mean age = 48.2 ± 16.0 years) from the IXI dataset were analyzed (IXI dataset, 2018). The age distribution of the subjects is summarized in Table 1. The 1,104 3T T1 weighted MP-RAGE MR images of 552 subjects were used. Before data analysis, all the scans were resampled to isotropic 1 mm resolution, and we named that native resolution.

**Table 1:** The amount of MRI acquisition subjects in each age range from the IXI dataset.

**Age range (years)**	**Num. subjects**
20–29	100
30–39	99
40–49	89
50–59	98
60–69	117
70–79	49
Total	552

**Figure 2:**
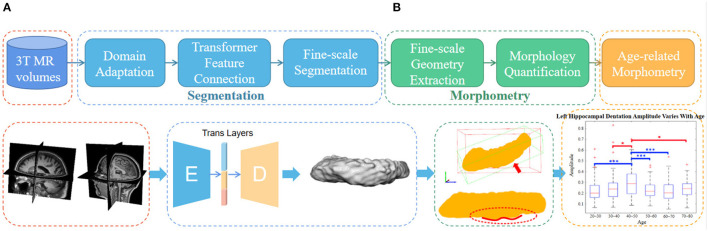
The overall flowchart of the proposed fine-scale segmentation and morphometry. **(A)** Hippocampus are segmented from the 3T MR images and the dentation and obtained from fine-scale segmentation. **(B)** After that, geometry features are extracted and age-based associations are explored. Dotted boxes of the same color indicate that they belong to the same content in the pipeline. Note that this only represents the overall flow of the manuscript. The detailed and high resolution figures for each portion here will be shown in the subsequent part.

As shown in the flowchart in Figure 2, the main pipeline consists of three parts. First, an automatic and robust segmentation algorithm was proposed to capture the fine-scale morphology of the hippocampus based on common 3T MR images. Then, the characteristic fine-scale dentation is extracted from the segmentation through a non-linear regressor. After that, the level of the dentations is quantitatively analyzed against age to identify temporal changes. In what follows, we detail all three major algorithm components.

### 2.1. Fine-scale semantic segmentation

The multi-atlas-based methods used in Chang et al. (2018) have the disadvantage of involving a large computation time when applied to a large-scale dataset for study. Therefore, we propose a new deep-learning-based fine-scale segmentation method to obtain fine-scale dentation of the hippocampus from 3T MR scans relating to 552 subjects. However, there are some serious issues to solve before such fine-morphological analysis can be efficiently applied to such a large cohort.

The first issue is that the large amount of image data used to segment the multi-atlas-based approach used in previous approaches, such as Chang et al. (2018), is too time-consuming to be practically useful. Toward this goal, the recent development of deep learning methods provides a promising alternative to multi-atlas approaches.

Second, the large IXI cohort analyzed does not have any manual annotation. We, therefore, need to utilize carefully validated annotation from the EADC-ADNI dataset (Apostolova et al., 2015; Boccardi et al., 2015; Frisoni et al., 2015) for training, and apply the trained model to the IXI dataset. This inevitably introduces a cross-dataset discrepancy between the training and testing images, and adequate domain adaptation is necessary.

Third, and most importantly, even the expert-curated hippocampus annotation in Apostolova et al. (2015), Boccardi et al. (2015), and Frisoni et al. (2015) does not capture the fine-scale hippocampus dentations and a deep learning model trained on such annotation is not capable. To perform the sub-pixel fine-scale morphometry, we have to depart from the constraint of the learned space and extend the segmentation to a much higher resolution level.

**Figure 3:**
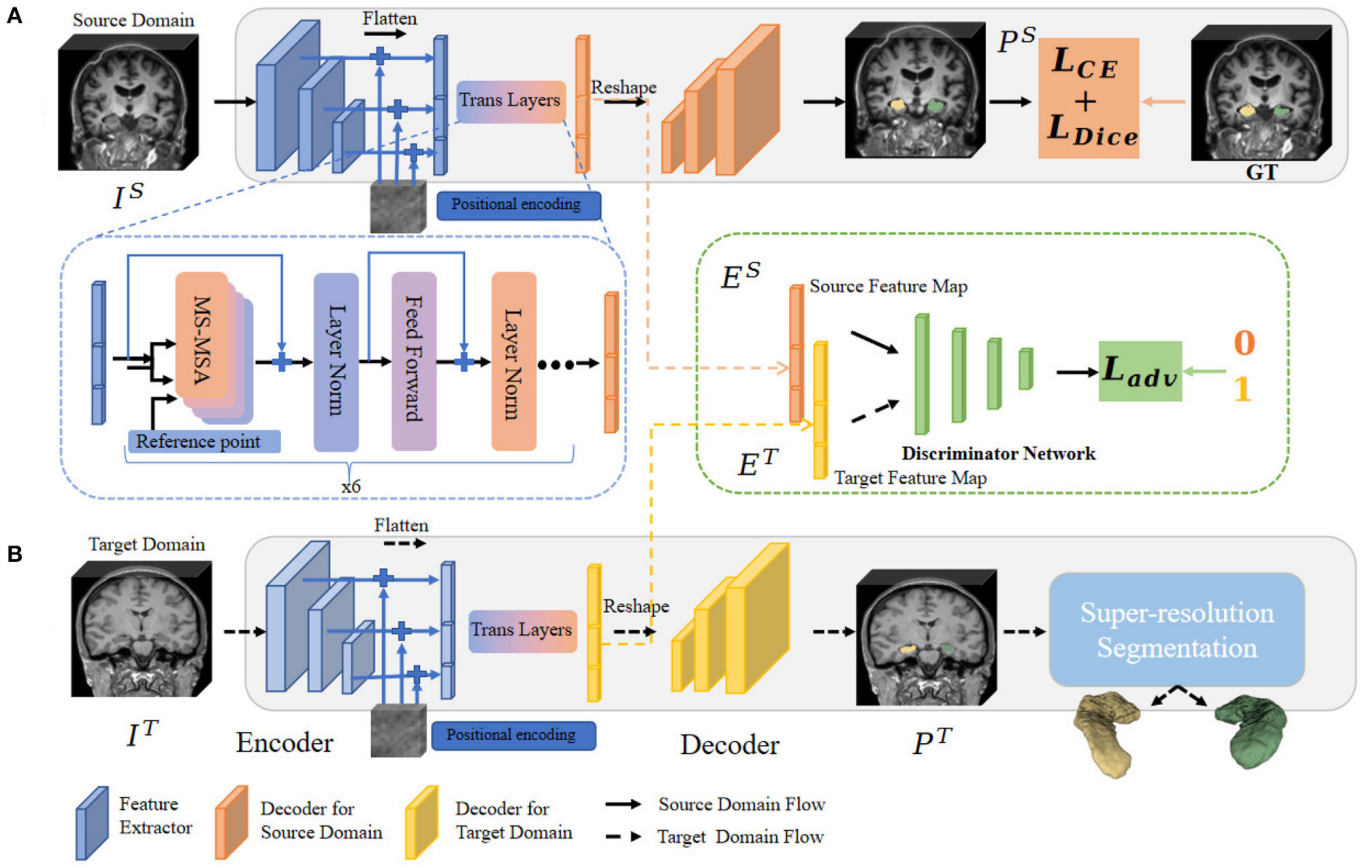
Overview of the fine-scale segmentation framework. The 3D MR images in the source and target domain pass through procedures **(A, B)**, respectively. In procedure **(A)**, CE and Dice Loss are computed based on source annotation to optimize the semantic segmentation framework. The multi-feature from different level layers also passes through the MSA layers and outputs the convergence feature. Next, images from the target domain were fed into procedure **(B)**. Before obtaining a fine-scale segmentation map, we adopt a module to constrain the extracted feature map between two different domains. The domain adaptation operation is illustrated in the block with a green edge. Finally, the possibility map from the target domain is forwarded through fine-scale segmentation (the blue block) to output the final annotation.

To address the above issues, in this sub-section, we propose the hippocampal domain adaption fine-scale segmentation method to capture the fine-scale hippocampus dentation structure from the clinically available 3T MR images. The algorithm pipeline is illustrated in Figure 3.

#### 2.1.1. Semantic segmentation

The deep-learning-based semantic segmentation method formulates the dentation annotation task as a pixel-classification problem. The core encoder-decoder framework consists of a stack of sequentially connected convolutional layers and long-range skip connections. The locations of the feature information in a higher layer are computed based on the locations of tensors of the next lower layers as they are connected through a layer-by-layer up-sampling operation. However, due to the locality nature of the convolution operation, the receptive field is limited along with the depth of layers and the size of the convolutional kernel. As a result, only higher layers with big receptive fields can model long-range dependencies in the vanilla encoder-decoder architecture. More recently, the multi-head self-attention mechanism (MSA) of the vision transformer shows a more effective strategy for learning long-range contextual information. As a result, we utilize a transformer-based MSA framework to overcome this limitation, which is motivated by Xie et al. (2021).

As shown in Figure 3, we bridge the transformer layer to the design of encoder architecture and aim to help engage lower and higher contextual features directly and capture the long-range dependency of pixels effectively. With such an encoder partition, the multi-scale features extracted from convolution are concatenated before being forwarded through MSA. However, Xie et al. (2021) sets the hidden size in residual blocks of the hierarchical encoder to 384 to keep the same hidden size in the feed forward network of MSA. At the same time, the small kernel size 3 used shows a lower capture ability, while the larger kernel size can capture dependencies between information units further away in the earlier layers. It only accepts inputs of the same size (48 × 192 × 192), which is not conducive to the segmentation of small organs, such as the hippocampus. In contrast to Xie et al. (2021), we squeeze the channels of the residual blocks to 192 as half of the original 384 channels and further adopt several groups of larger kernel-sized convolutions to expand the dependencies capture ability of inner-place units. Moreover, we engage the last three layers of contextual feature output from the encoder together to get finer-scale spatial information. In short, we improve by replacing the channel complexity with spatial complexity. We also modified the size limit of the input to be able to take a smaller size of 64^3^ than 48 × 192 × 192 in dimensions 2 and 3 as input and focus more on the target hippocampal region. In the next step, these extracted features are passed to the MSA layers to aggregate hierarchical long-range dependency.

It should be mentioned that there is a mismatch between the 3D image tensor and the 1D sequence when bridging the transformer layer. As linear projection processes the information in a sequence-to-sequence manner, the feature maps produced by the encoder from every stage must be flattened into a 1D sequence before feeding into transformer layers. Also, it has to face the problem of losing spatial information when it is being flattened. So we add the 3D positional encoding sequence to supplement position information to solve this problem. Furthermore, to improve the computational efficiency, we utilize the set of key sampling locations (denoted as *r*_*p*_) in the image around the reference location. As a result, the MSA layers can be formulated as:


(1) $$\begin{array}{l} MSA\left({\left\{f_{l}\right\}}_{l=1}^{L},z_{q},r_{p}\right)=\text{Ψ}\left(Concat\left(h_{1},h_{2},\ldots ,h_{N}\right)\right) \end{array}$$

where *N* is the number of the heads (denoted as *h*_*i*_ and set as 6), and flattened feature maps ${\left\{f_{l}\right\}}_{l=1}^{L}$ are extracted from the *L* stages of the left encoder. *z*_*q*_ is the feature representation of query *q*, which is gotten from ${\left\{f_{l}\right\}}_{l=1}^{L}$ and position embedding feature, Ψ(·) is the Linear projection operation to weight and aggregate the features.

In the Decoder part, the output sequences of transformer layers are separated and reshaped according to the size of feature maps from the encoder at each stage. The processed features from each stage are then concatenated with processed features from the deconvolutional layers of the preceding stage. Finally, they are fed into a residual block followed by a 1 × 1 × 1 convolutional layer with a proper activation function (softmax) for computing the segmentation probabilities of the hippocampus.

To efficiently illustrate the workflow, we denoted the semantic segmentation framework as *S*. It takes the cropped sub-volumes of image volumes from Source Domain (denoted as *I*^*s*^:Ω^3^⊂ℝ^3^) as input, and generates an output of the same shape (denoted as *P*^*S*^:Ω^3^⊂ℝ^3^). Plus, all these volumes were resampled to isotropic of 1 mm^3^ before segmentation. The corresponding annotation of $I_{i}^{s}$ are also denoted as *L*^*s*^:Ω^3^⊂ℝ^3^, Ω^3^ → 0, 1, 2. In order to optimize *S* and get better parameter *W*_*S*_, we utilize the segmentation loss $L_{s}$, which is defined as:


(2) $${ℒ}_{s}=\lambda_{1}*\underset{{ℒ}_{ce}}{\underset{︸}{\frac{1}{N}\sum_{i=1}^{N}-y_{ij}\log\left(p_{ij}\right)}}+\underset{{ℒ}_{dice}}{\underset{︸}{\frac{1}{N}\sum_{c=1}^{N}-\frac{2\sum_{i}p_{ij}y_{ij}}{\sum_{i}p_{ij}+\sum_{i}y_{ij}}}}$$

where *p*_*ij*_ and *y*_*ij*_ refer to the segmentation predicted probability and corresponding category segmentation for voxel *i*, *j*. *N* means the voxel number. The segmentation loss function can be minimized end-to-end by getting optimized *W*_*S*_. Finally, the output channel of the network is set as 3, for the left, the right hippocampus, and the background. This semantic segmentation framework is intended for images from the ADNI dataset with observed distribution, and the next step is to address the problem of obtaining hippocampal annotation for images from the IXI dataset with unobserved distribution.

#### 2.1.2. Image normalization through domain adaption

The MR images of the large IXI cohort to be analyzed are acquired from different machines and are of different protocols from the training MRI cohort where the hippocampus is labeled. Since MR images across machines do not share reference voxel values, the training images may have different intensity values and/or texture patterns from the testing ones.

As a result, we have to normalize the distribution of the images from the IXI dataset (the target domain) with those in the training and validation ADNI datasets (the source domain). To that end, we train our segmentation model with a discriminator network to make the adaption between the two sets.

Denote the two sets of images from the source and target domains as *I*^*s*^ and *I*^*t*^, respectively. We forwarded the source image *I*^*s*^ to the semantic segmentation network *S* and calculate the difference between output and annotations for an optimal *S*. Then, we predicted the segmentation output *P*^*t*^ for the target image *I*^*t*^. Since our goal is to make segmentation predictions *P*^*s*^ of source and *P*^*t*^ of target images close to each other, we used these two predictions from the segmentation framework as the input to the discriminator *D* to distinguish whether the input is from the source or target domain.

Optimizing the adversarial loss on the target prediction, the network propagates loss gradients from *D* to *S*, which encourages *S* to generate similar segmentation distributions in the target domain to the source prediction. With the proposed method, we formulate the adaptation task containing discriminator loss functions:


(3) $$\begin{array}{l} L_{total}=L_{seg}+\lambda_{adv}L_{adv} \end{array}$$

where $L_{seg}$ is the semantic segmentation loss using ground truth annotations in the source domain, and $L_{adv}$ is the adversarial loss that adapts predicted segmentations of target images to the distribution of source predictions. The λ_*adv*_ is the weight used to balance the two losses.

For the discriminator, we use an architecture similar to Tsai et al. (2018) but utilize the extracted feature from transformer layers and the final softmax segmentation possibility map to explore more spatial information. Furthermore, only one discriminator is utilized in our framework. The discriminator network consists of convolution layers followed by an adaptive average pooling unit and a full connection layer for the binary classification as illustrated in Figure 3 (green block). The discrimination cross-entropy loss can be written as:


(4) $$\begin{array}{l} L_{adv}\left(E\right)=-\sum \left(\left(1-z\right)\log\left(D\left(E\right)\right)+z\log\left(D\left(E\right)\right)\right) \end{array}$$

For discrimination, *z* is set to 0 if the sample is gotten from the source domain and *z* is set to 1 if the sample is from the target domain.

#### 2.1.3. Fine-scale segmentation

Although a rich amount of deep-learning based hippocampus segmentation schemes exist, one of their shortcomings is that at the native image resolution the fine dentation morphology is not captured in the manual annotation. Moreover, as the deep-learning based methods depend more on the training annotation, it is apparent that if certain shape features do not exist in the training set, it is unlikely they will be captured accurately in the external testing images.

On the other hand, the probability map of the hippocampus obtained above contains valuable information about the approximate morphology. Since we aim to extract the fine morphology, it is valuable to escape the realm learned by the deep learning framework and explore the fine-scale morphology features unseen in the training images. This is detailed below.

To refine the surface of the hippocampus, we employ the fine-scale method (denoted as *SR*(·)) to fine-tune the probability map *M*^*T*^:Ω^*s*^ → [0, 1] from *S* at the native resolution. However, when such a data-driven method is carried out in a high-resolution space, it will consume more than 100 times in computer memory and complexity compared to native resolution. To reduce such a burden and make the computation practical, we only handle the region of interest focal to the hippocampus according to the possibility map *M*^*T*^ of target domain image *I*^*T*^ to get *R*^*T*^. This step of cropping is performed automatically by the program. By doing this, we can perform segmentation on a sub-millimeter level morphological feature contained in the grayscale information and get the final result *S*^*sr*^. The operation can be denoted as:


(5) $$\begin{array}{l} S^{sr}=SR\left(Crop\left(M^{T}\right)\right) \end{array}$$

More explicitly, we get the sampled observation in isotropic 0.2 mm/voxel resolution by the factor *H* being set as 5 and up-sampled the images through convex 3D interpolation while balancing the consumption and efficiency in the morphology study. To construct the hippocampus in high resolution space with fine location, first, we define the high confidence region as C: = {*x*∈Ω^*s*/*H*^:*M*^*T*^(*x*)>η}, where higher η∈[0, 1] values indicates the voxel belonging to hippocampus with higher confidence and Ω^*s*/*H*^ is the new images after *H* times cubic spline up-sampling. However, such a strongly constrained *C* does not make full use of the hippocampal surface context information of images in the high image space, which might even crudely omit some dentations.

To address this issue, we used the following variational approaches to refine the hippocampal surfaces. We denote the family of evolving surface as ζ⊂ℝ^3^, ζ = ∂*C*, and for surface optimization, we define an energy functional as:


(6) $$E(\zeta ):\text{​​}=-\int_{x\text{ in }\zeta }\alpha M^{T}(x)dx+\beta \int_{\zeta }dA$$

where the *x* traverses the space inside the closed surface ζ, and the joined $\underset{\zeta }{\int }dA$ is the total surface area. The α and β are the positive weight. Calculating regional statistic force and edge-based force, the flow of the surface is controlled by the partial differential equation below:


(7) $$\frac{\partial \zeta (p,t)}{\partial t}=[L((p,t))-\alpha M^{T}(\zeta (p,t))+\beta \nu (p,t)]V(p,t)$$

where **V** is defined as the inward unit normal vector field on ζ, ***p*** is the spatial parameterization of surface and ν is the mean curvature of the surface. In Equation (7), Laplacian of Gaussian function (LoG) is defined as *L*((***p***, *t*)) for edge based force. To balance the force of edge evolution, the joined term on the right of LoG is the regional statistic force. The surface optimizing Equation (7) does not necessarily reside in the learned space of the neural network. This means it escapes from the learned space, which does not have the fine morphology, and the surface evolves and converges to the locations that process strong edge appearance and close to the probability map *M*^*T*^ with high confidence (control by setting η value).

Both the deep-learning and fine-tuning processes above are fully automated. As a result, the final surface will not only achieve high local similarity measures such as the Dice coefficient but will also successfully capture the fine-scale hippocampal dentations, which is the critical shape feature for subsequent morphology studies.

### 2.2. Dentation feature extraction and analysis

The goal of dentation analysis is to quantitatively explore the denotational shape variation between different groups. To this end, we first extract the dentation region by projecting the shape to a proper plane. Then, the dentation could be modeled as sinusoidal curves, whose parameters are obtained by non-linear fitting. Once the parameters of the curves have been found, the dentations across different groups are compared.

**Figure 4:**
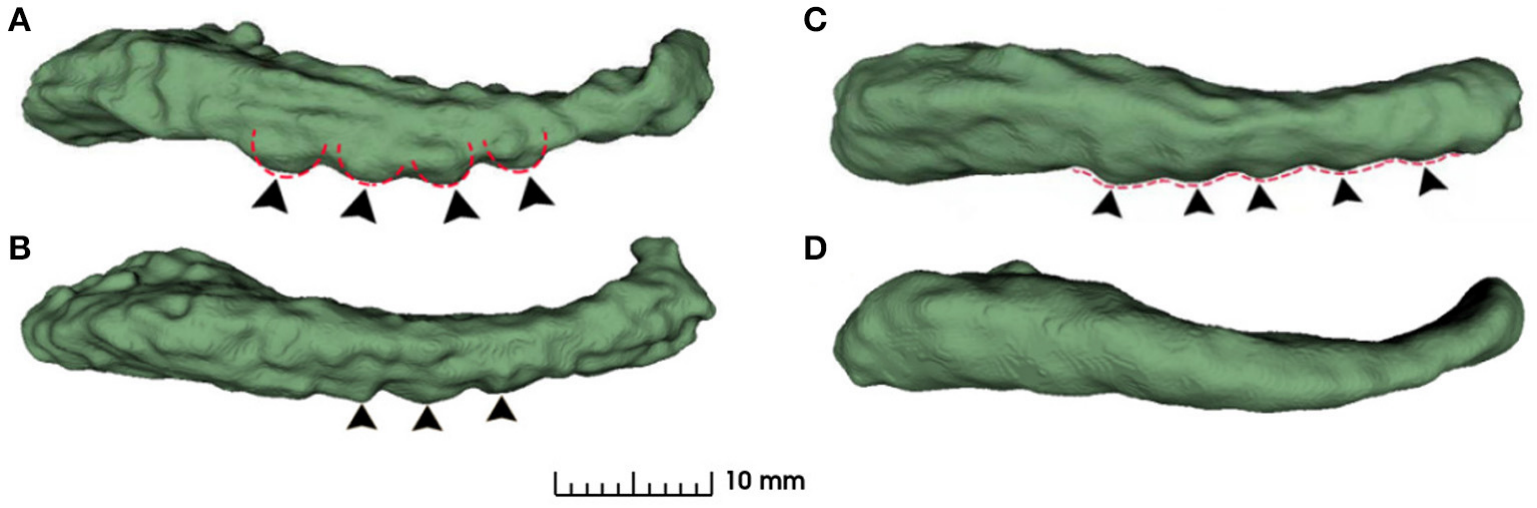
Examples of variation in degree of dentation. **(A)** Depicts a high degree of dentation with many prominent, or arciform dentations. **(B)** Depicts a few arciform dentations. **(C)** Shows few, less prominent sinusoidal dentation. **(D)** Shows a hippocampus with no apparent dentation. Arrowheads indicate individual dentation. Dashed red lines illustrate the contour of arciform **(A)** and sinusoidal **(C)** dentation.

As can be seen from Figure 4, the dentations reside on the inferior surface of the CA1 section and are one or more relatively parallel ridges. Based on such observation, if one could project the 3D dentation structure along its ridge, the resulting 2D silhouette should have a sinusoidal appearance. It is workable to only capture the magnitude and frequency of such sinusoidal waves to characterize the dentations. Following the ideas above, the proposed method contains the following steps.

#### 2.2.1. Point cloud representation

The fine-scale segmentation method provides a very detailed extraction of the hippocampal structure and allows a detailed analysis of the dentation structure. Following the ideas above, we first project the 3D shape to a plane that optimally reveals the dentation in the 2D plane. To aid the projection step, we represent the extracted hippocampus using a point cloud. Following Gao and Tannenbaum (2010), the point cloud is a collection of data points defined by a given coordinate system, which carries the morphological information of hippocampal structure. The segmented hippocampus region can be denoted as a binary image *J*:ℝ^3^ → 0, 1. *J* can therefore be considered as a probability density function (pdf) of a random variable which uniformly distributed in the hippocampus region. Next, we extract samples from such a pdf. Due to the irregular shape of *J*'s support, we employ the rejection sampling for the sample extraction. As a result, each hippocampus is represented as a cloud of points $X=x_{i}\in {\mathbb{R}}^{3}$ that are further processed in the subsequent sections.

#### 2.2.2. Medial axis representation

Looking laterally, the dentation features (or lack thereof) are evident underneath the gyrus region. One needs to project the 3D shape to the correct 2D view, identify the inferior boundary, and then quantitatively represent the dentation feature for comparison across ages. In this context, principal component analysis (PCA) is a suitable and effective linear dimension reduction technique to serve the purpose of extracting the AP axis and the inferior surface of the hippocampus.

In practice, PCA projects the data points to the subspace, which maximizes the variance to keep the structure of interest in the volume as much as possible. The first few eigenvectors that yield the largest eigenvalues often explain most of the variance in the data. The shape of the hippocampus has the longest axis in the AP direction. After that, the lateral width of the hippocampus is about 3 cm whereas the thickness in the superior-inferior direction is the smallest, which is less than 1 cm. As a result, when performing the PCA on the points in the hippocampus, the eigenvector corresponding to the largest eigenvalue is expected to be roughly the AP direction but slightly tilted up. Following that, the second mode should be in the left-right direction and the third eigenvector should be perpendicular to the “sheet” of the hippocampus.

**Figure 5:**
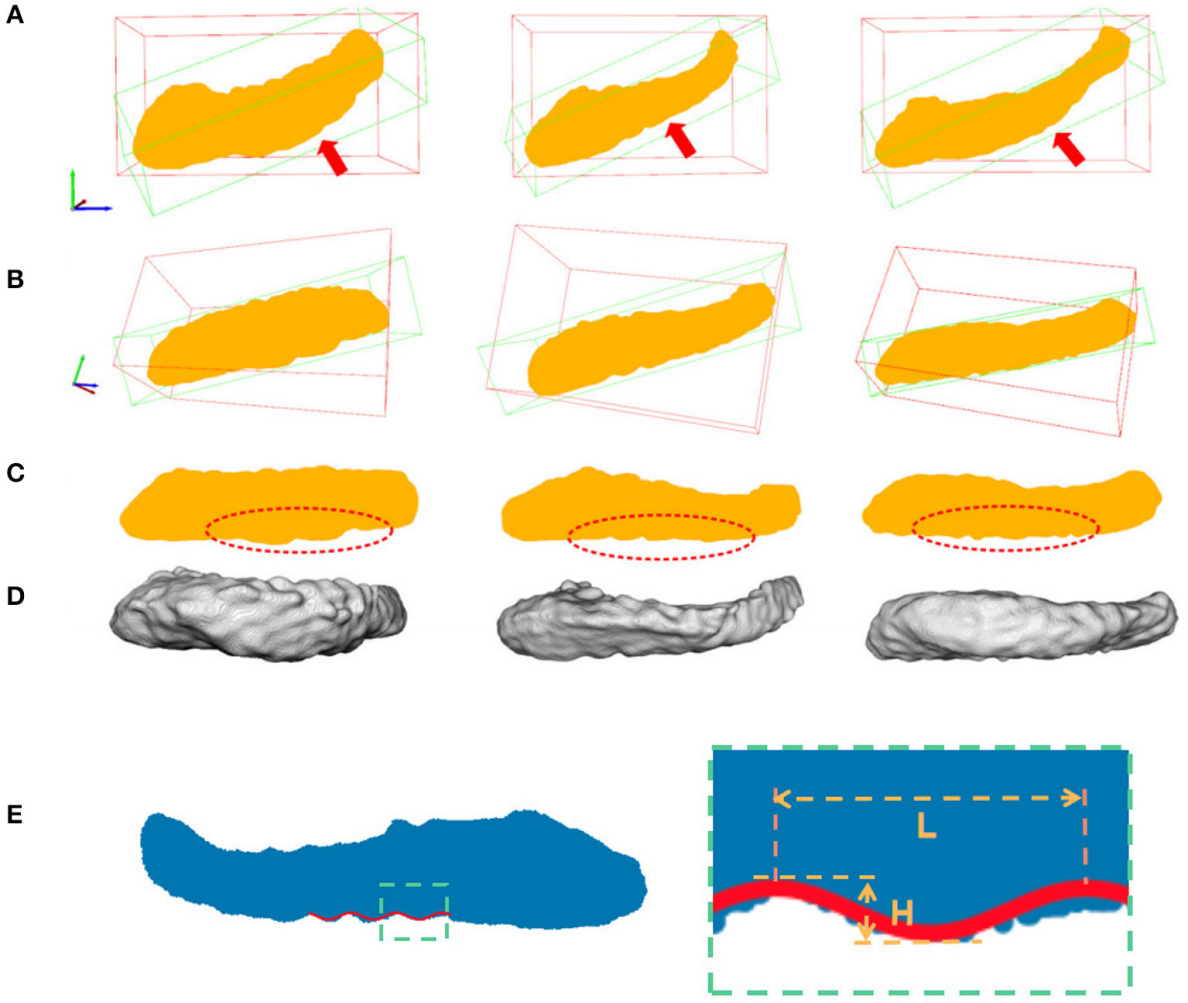
Hippocampal 3D point cloud representation, with the resulting plane of interest. Rows **(A, B)** show the hippocampal results from fine-scale segmentation. Row **(C)** shows the corresponding point cloud representation of hippocampus. The red arrows showed the second major sight of view in 3D space. The red bounding boxes are axis-aligned bounding boxes of point cloud and the green bounding boxes are oriented bounding boxes based on the PCA of their convex hull. Row **(D)** are results after dimension reduction processing and dentation can be seen in it. Row **(E)** shows measurements of the height and width of hippocampal dentition after PCA. H represents the peak and trough distance of the curve, and L represents the length of one bump of the curve.

As a result, if we project all the 3D points in the hippocampus along the second direction onto the plane spanned by the first and third eigenvectors, we could observe the dentations clearly in the 2D view. This is shown in Figure 5.

#### 2.2.3. Sinusoidal dentation modeling and fitting

As shown in Figure 5, hippocampus dentation on its inferior surface is observed to have many arciform or sinusoidal prominence in the dotted red circle. A similar variation pattern in 3D is reflected in the 2D geometry after the projection. This variation of appearance can be approximated by a sinusoidal fitting model, which allows for a quantitative description by measuring dentation with two parameters of magnitude and frequency. The core of the quantitative analysis is to find model parameters that can reflect the prominence of dentation in the hippocampal sub-region, which has inter-subject variation. In this work, a non-linear fitting model was established to explore and characterize this simple dentation variation. We compute the model parameters that lead to an optimal adaptation of the variation to the set of observations. Specifically, we fit a sinusoidal function to the silhouette of the inferior surface of the hippocampus and measure the parameters of the sinusoidal function. Mathematically, we build a two-dimensional Cartesian coordinate for each hippocampus. This coordinate has its origin at the center of mass of the hippocampus. Next, two axes are pointing to the eigenvectors with the largest and smallest eigenvalues from the PCA method, respectively. Visually, this forms a plane cutting through the hippocampus vertically along its major axis. All points in the hippocampus are projected onto this plane, forming a 2D region as shown in row 3 of Figure 5.

The silhouette of the inferior hippocampal surface is therefore denoted as a function in this coordinate system. The sinusoidal fitting is cast as an optimization problem:


(8) $$J\left(A,w,\phi ,b\right):\text{​}=\int_{x}\left(y\left(x\right)-{\left[A\sin\left(wx+\phi \right)+b\right]}^{2}\right)dx$$

In Equation (8), there are four fitting parameters: amplitude (***A***), frequency (***f*** = ***w***/2π), phase (**ϕ**), and bias (***b***). Among them, amplitude and frequency are the two key parameters that describe the height and the density of dentations.

For ease of understanding, we will obtain the height and width of the hippocampal dentation and display it graphically. As shown in Figure 5E, the height *H* is twice the amplitude ***A***, so *H* = 2***A***, and the hippocampal bump width can be expressed as *L* = 1/***f***. Therefore, the goal is to find parameters to minimize *J* to obtain the optimal parameter magnitude and frequency and this is a non-linear optimization problem.

In order to address this non-linear optimization problem, simulated annealing (SA) (Khachaturyan et al., 1981) was employed to find the optimal parameters ***A***, ***w***, **ϕ**, ***b***. SA is a probabilistic approach for getting the proximate global optimum of a given non-linear function. Compared with the general greedy algorithm, the SA introduces random factors, which may accept a solution worse than the current solution with a certain probability. This means that SA is able to jump out of the local optimal solution and approximate the global optimal solution.

The following annealing criteria are used to allow for accepting a “worse” solution:


(9) $$\begin{array}{l} {\text{e}}^{-\Delta D/T}>R\left(0,1\right) \end{array}$$

where Δ*D* is the difference of cost implied by the balance, the temperature is initialized high and gradually “cool” to simulate the heating process, and *R*(0, 1) is randomly distributed on [0,1].

### 2.3. Experiments and evaluations

#### 2.3.1. Experimental setup

For this study, obtaining accurate fine-scale segmentation results is the prerequisite for accurate longitudinal analysis. It is therefore critical to evaluate the fine segmentation performance of the proposed framework. To that end, the proposed framework was compared against the following state-of-the-art (SOTA) techniques: Hippodeep (Thyreau et al., 2018) proposed a CNN trained on hippocampal segmentation from multiple cohorts including 2,500 T1 MR scan images. HippMapp3r (Goubran et al., 2020) proposed and trained a 3D CNN using 259 bilateral manually delineated segmentation acquired at multiple sites on different scanners. In addition, to acquire better performance of encoder and decoder, we utilized the based segmentation framework proposed in Xie et al. (2021). Therefore, it is also included in comparison experiments.

We performed our experiments on the two datasets. The EADC-ADNI dataset (Apostolova et al., 2015; Boccardi et al., 2015; Frisoni et al., 2015) containing 41 manually labeled subjects was randomly divided into a training group (*N* = 30) a validation group (*N* = 11). IXI dataset (2018) contains 552 subjects and all of them were utilized as test groups. The proposed frameworks were trained on the training group, the validation group, and the test group for evaluation. To evaluate the performance of hippocampal segmentation on the IXI dataset, we randomly selected 150 subjects from the IXI dataset for testing. To obtain the corresponding manual annotation and to reduce the workload, we first obtained the annotation with the FSL-FIRST (Patenaude et al., 2011) as the initial segmentation and then manually corrected the segmentation results to serve as the correct manual annotation.

#### 2.3.2. Evaluation metrics

To evaluate the hippocampal segmentation results against expert manual annotation, quantitative measurements of Dice similarity coefficient (DSC), Jaccard, Precision, Recall (shown in Equation 10), Hausdorff distance, and 95*th* percentile of the distance (95% HD; shown in Equations 11, 12) were used: all of which are standard metrics and are defined as:


(10) $$\begin{array}{l} Recall=\frac{TP}{TP+FN},\;Precision=\frac{TP}{TP+FP},\\ \;DSC=\frac{2TP}{2TP+FP+FN},\;Jaccard=\frac{TP}{TP+FP+FN} \end{array}$$

(11) $$\begin{array}{l} \;h_{95}\left(X,Y\right)=K_{x\in X}^{95}\underset{y\in Y}{\min}|x-y|,\\ HD_{95}\left(X,Y\right)=\max\left(h_{95}\left(X,Y\right),h_{95}\left(Y,X\right)\right) \end{array}$$

(12) $$HD\left(X,Y\right)=\max(\underset{x\in X}{\max}\underset{y\in Y}{\min}|x-y|,\underset{y\in Y}{\max}\underset{x\in X}{\min}|y-x|)$$

In it, *TP* denotes the true positive, which means the predicted voxel coincides with the ground-truth; *FP* denotes the false positive, which means the predicted voxel falls outsides the annotation region of ground-truth; *FN* denotes the false negatives, which means the predicted background voxel is inside the ground-truth. *h*_95_(*X, Y*) is the 95*th* ranked minimum Euclidean distance between boundary points in *X* and *Y*. While DSC captures a volumetric-overlapping between the segmentations and the reference standard, the HD and 95*th* HD measure the point-wise distance.

#### 2.3.3. Network training

All experiments were implemented in Python3.7 Pytorch backend (version 1.9) and trained on an NVIDIA RTX A6000 graphics card with 48GB of memory. In the training phase, all the network architecture was trained on the EADC-ADNI dataset with hippocampal annotation for 2,000 epochs with a batch size of 8. In the discriminator network, the convolutional kernel size is set as 4 and the stride is set as 2. To balance the training loss λ_*ce*_ and λ_*dice*_, the parameter λ_1_ is set as 0.1. And following (Tsai et al., 2018), λ_*adv*_ is set as 0.001. For optimization, the Adam optimizer was adopted with a learning rate of 10^−4^ for gradient update. As the hippocampus occupied only a small region in the brain scan images, to focus on the local feature around the hippocampus, each labeled MRI volume was randomly cropped to 64 × 64 × 64 voxels for model input as mentioned in Tian et al. (2021).

The sampled subjects from these two datasets are acquired from the different MR scanners at different study sites. This results in different voxel spacings, directions, and intensity ranges. Hence, before training, all the images were resampled to an isotropic voxel spacing of 1 mm/pixel according to the subjects from the EADC-ADNI dataset using the SimpleITK toolkit.

#### 2.3.4. Examining group differences across age groups

To quantitatively measure the dentation within difference groups, we investigated the above two measurements (frequency and amplitude) in each age group and made comparisons across the groups. Finally, for quantitative analysis, we tested for statistically significant differences among age groups by performing student *t*-tests.

## 3. Results

In this section, we present and compare the hippocampal segmentation results of various methods on two datasets. Furthermore, to verify the robustness of the method, we also apply the framework to 7T MR scans to obtain fine segmentation results. After that, based on the proposed fine-scale segmentation method, we performed the fine-scale hippocampal morphometry study on a group of 552 healthy subjects.

Section 3.1 shows the training and validation results of different segmentation methods on the EADC-ADNI dataset. The results of the proposed fine-scale segmentation algorithm are presented in Section 3.2. Section 3.3 shows the obtained segmentation results based on the proposed framework and manual annotation in 7T scans. Finally, we used the fine-scale hippocampal segmentation from Section 3.2 to perform morphological analysis of the hippocampus of healthy subjects across different age groups, in Section 3.4.

### 3.1. Segmentation evaluation of EADC-ADNI dataset

#### 3.1.1. Segmentation results at native resolution

In this section, we demonstrate the comparison between the proposed method and some of the SOTA segmentation algorithms on the EADC-ADNI dataset at the native image resolution. The results of ablation experiments of the proposed framework are also presented.

**Figure 6:**
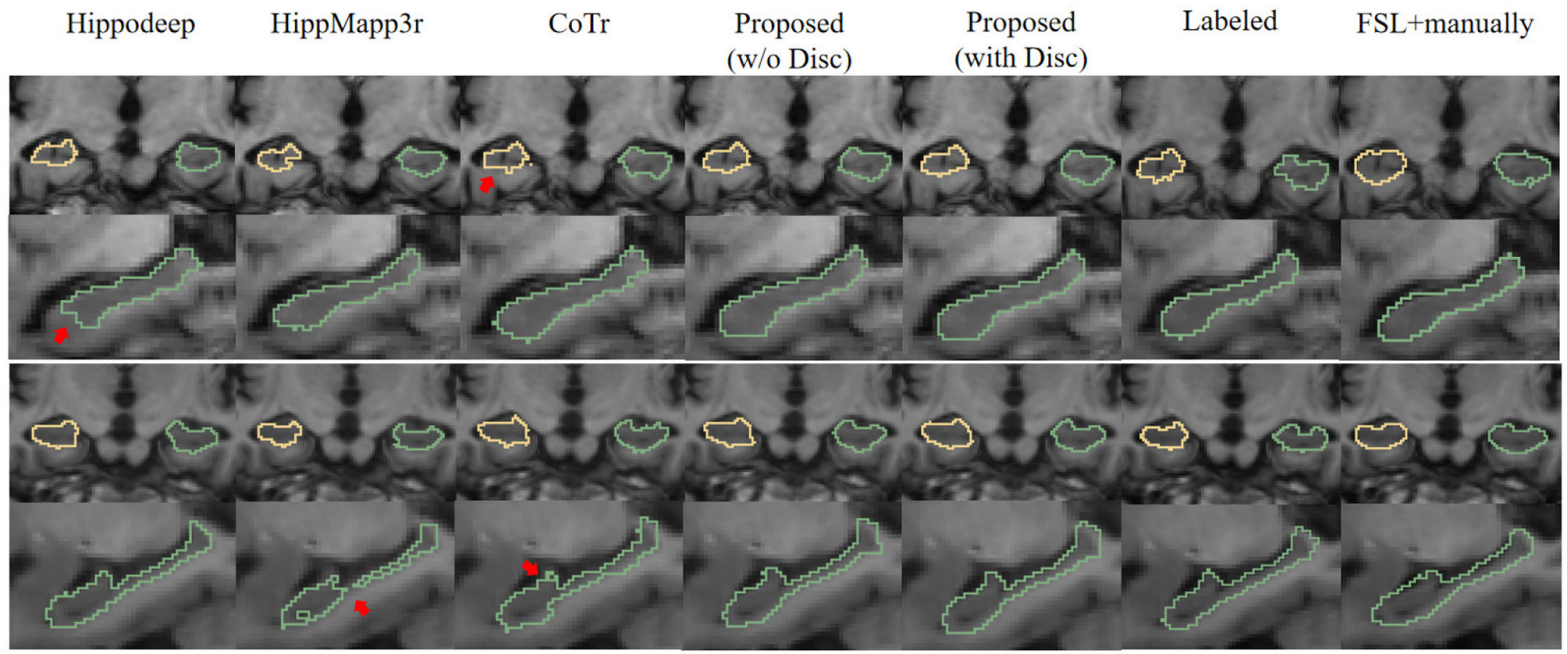
Visual contour and comparison of different segmentation methods on two examples subjects. The first and second rows are from the first subject, and the third and fourth rows are from the second subject. The first and third rows display the coronal slices of the left and right hippocampus, while the second and fourth rows display segmentation in sagittal slices. Manually traced ground truth is displayed in the penultimate column on the right. Annotation getting from FSL and manual correction results are shown in the right most column. The reason for including this “FSL+manually” column is to enable the consistent comparison of the dataset without too many manual annotations: see text in Section 3.1.2 for details. The red arrows indicate the over- or under-segmentation error.

Figure 6 is a visualization of the algorithm results and manual annotations. It shows that the HippoDeep algorithm is missing some parts of the hippocampal head. This may be caused by the fact that the amygdala is sharing a very similar appearance with the hippocampus. As a result, a conservative algorithm would try to avoid leaking into the amygdala region, resulting in slight under segmentation of the hippocampal head. Along a similar vein, the HippMapp3r algorithm is missing some parts of the subiculum. The other algorithms are giving relatively satisfying results, except that the CoTr is missing bits of the CA3.

Table 2 shows the quantitative analysis results of the hippocampal predictions of various methods compared with the original labels of the EADC-ADNI dataset.

**Table 2:** Performance comparison of segmentation results of EADC-ADNI dataset in native resolution using different methods.

**Framework**	**Left_Right**	**DSC↑**	**Jaccard↑**	**Recall↑**	**Precision↑**	**HD95 (mm)↓**	**HD (mm)↓**
Hippodeep (Thyreau et al., 2018)	Right	0.827 ± 0.027	0.706 ± 0.038	0.792 ± 0.052	0.868 ± 0.016	1.63 ± 0.49	4.23 ± 1.08
	Left	0.831 ± 0.024	0.712 ± 0.034	0.799 ± 0.052	0.87 ± 0.026	1.70 ± 0.45	4.08 ± 1.15
HippMapp3r (Goubran et al., 2020)	Right	0.808 ± 0.012	0.678 ± 0.017	0.702 ± 0.02	**0.953** **±** **0.012**	1.89 ± 0.26	4.34 ± 0.88
	Left	0.812 ± 0.018	0.684 ± 0.026	0.71 ± 0.029	**0.949** **±** **0.012**	1.63 ± 0.27	3.59 ± 0.61
CoTr (Xie et al., 2021)	Right	0.862 ± 0.012	0.757 ± 0.018	0.878 ± 0.027	0.847 ± 0.017	1.47 ± 0.17	3.36 ± 0.51
	Left	0.862 ± 0.01	0.758 ± 0.015	0.892 ± 0.028	0.835 ± 0.024	1.44 ± 0.09	3.42 ± 0.39
Proposed (w/o Disc)	Right	0.873 ± 0.008	0.774 ± 0.013	0.893 ± 0.015	0.854 ± 0.019	1.41 ± 0.01	**3.32** **±** **0.59**
	Left	0.873 ± 0.011	0.774 ± 0.017	0.907 ± 0.02	0.842 ± 0.022	1.41 ± 0.16	**3.09** **±** **0.44**
Proposed (w Disc)	Right	**0.876** **±** **0.008**	**0.779** **±** **0.012**	**0.906** **±** **0.018**	0.848 ± 0.018	**1.41** **±** **0.01**	3.38 ± 0.63
	Left	**0.875** **±** **0.012**	**0.778** **±** **0.019**	**0.910** **±** **0.024**	0.844 ± 0.027	**1.37** **±** **0.20**	3.35 ± 0.69

The upward arrow indicates that higher values are better and the downward arrow indicates that lower is better. The best results are marked in bold.

First, comparing the comparison of the segmentation results obtained from different segmentation algorithms, the proposed framework has a maximum improvement of 6.8% in DSC metrics (left hippocampus, compared to HippMapp3r) and a minimum improvement of 6.3% (left hippocampus, compared to HippMapp3r). HippMapp3r has the highest average precision. But this may be because it is more likely to under-segment the hippocampus, which is consistent with the visual appearance in the bottom panel in Figure 6. It is noticed that the HD95 and HD of the CoTr framework are better than those of the proposed method. However, CoTr sometimes suffers under-segmentation in the hippocampus region with lower DSC performance than that of ours in the experiment.

Second, to verify the effectiveness of each component of the proposed framework, two ablation experiments are conducted and the results are shown in Table 2. (1) The proposed framework without the discriminator is improved based on CoTr. Compared to CoTr, our proposed framework without the discriminator part achieved a higher overlap evaluation score and lower segmentation error around the edge of the hippocampus (increased by 0.3 mm in HD metric on the left). (2) Comparison of the models with and without the discriminator: we found that the former achieves the best performance in most evaluation metrics. In summary, these two ablation experiments demonstrate the effectiveness of our proposed framework for improving the segmentation performance of the hippocampus at the native resolution.

#### 3.1.2. Constructing consistent hippocampus annotations between two datasets

Although the EADC-ADNI dataset contains the 3D manual annotation, the IXI dataset, unfortunately, does not. Therefore, to conduct a consistent comparison between the two datasets, we have to create a consistent reference for both. Since directly labeling the hippocampus would be time-consuming, following (Liu et al., 2020), we used the results from FSL-FIRST (Patenaude et al., 2011) as the initial segmentation and make manual corrections afterward. This created consistent 3D reference annotations for the two datasets. Next, they are collectively named FSL+manually labeled.

Table 3 presents the quantitative analysis results of the hippocampal segmentation of various methods compared with the FSL+manually label of the EADC-ADNI dataset. As can be seen, the proposed method can obtain the highest DSC metric. Moreover, the highest segmentation accuracy was obtained for both sides' hippocampi measured by the Jaccard, Recall, and HD95 metrics, similar to the case in Table 2. Although the DSC of our proposed method in Table 3 are approximately 3% lower than those in Table 2, this reduction is also observed in the other segmentation models. The performance of these segmentation models on the other metrics also shows a consistent change from Table 2 to Table 3, with Recall decreasing and Precision increasing. Moreover, the evaluation results in Table 2 are higher than those in Table 3 because in Table 2 those annotations for validation and training are drawn manually from the same group of annotation experts. Therefore, a reduction in the evaluation metrics is caused by the variability of the two different manual annotations.

**Table 3:** Quantitative comparison of segmentation results obtained by different segmentation methods with results from FSL+manual annotation on EADC-ADNI dataset.

**Framework**	**Left_Right**	**DSC↑**	**Jaccard↑**	**Recall↑**	**Precision↑**	**HD95 (mm)↓**	**HD (mm)↓**	**2D HD (mm)**
Hippodeep (Thyreau et al., 2018)	Right	0.800 ± 0.033	0.668 ± 0.044	0.715 ± 0.062	0.918 ± 0.054	2.13 ± 0.39	3.92 ± 0.76	2.12 ± 0.64
	Left	0.812 ± 0.025	0.684 ± 0.035	0.733 ± 0.05	0.915 ± 0.045	1.91 ± 0.31	3.82 ± 0.70	1.91 ± 0.43
HippMapp3r (Goubran et al., 2020)	Right	0.750 ± 0.026	0.60 ± 0.033	0.612 ± 0.04	**0.970** **±** **0.022**	2.38 ± 0.28	4.49 ± 0.74	2.41 ± 0.52
	Left	0.755 ± 0.022	0.607 ± 0.028	0.624 ± 0.031	**0.956** **±** **0.026**	2.27 ± 0.21	4.31 ± 1.01	2.14 ± 0.49
CoTr (Xie et al., 2021)	Right	0.820 ± 0.017	0.695 ± 0.024	0.752 ± 0.036	0.904 ± 0.029	1.99 ± 0.19	3.86 ± 0.42	1.65 ± 0.44
	Left	0.820 ± 0.019	0.696 ± 0.027	0.779 ± 0.025	0.868 ± 0.04	1.95 ± 0.26	**3.84** **±** **0.83**	1.78 ± 0.55
Proposed (w/o Disc)	Right	0.834 ± 0.012	0.715 ± 0.017	0.79 ± 0.026	0.884 ± 0.034	1.99 ± 0.18	4.12 ± 0.65	1.52 ± 0.38
	Left	0.827 ± 0.021	0.705 ± 0.03	0.803 ± 0.021	0.854 ± 0.04	1.94 ± 0.39	4.06 ± 0.95	1.70 ± 0.53
Proposed (w Disc)	Right	**0.847** **±** **0.013**	**0.734** **±** **0.019**	**0.810** **±** **0.029**	0.888 ± 0.029	**1.81** **±** **0.28**	**3.83** **±** **0.66**	**1.24** **±** **0.31**
	Left	**0.837** **±** **0.020**	**0.72** **±** **0.029**	**0.813** **±** **0.026**	0.864 ± 0.04	**1.85** **±** **0.42**	3.89 ± 1.55	**1.55** **±** **0.56**
Proposed (SR)	Right	0.866 ± 0.019	0.765 ± 0.029	0.823 ± 0.044	0.918 ± 0.027	1.49 ± 0.22	3.41 ± 0.84	1.16 ± 0.32
	Left	0.863 ± 0.016	0.759 ± 0.024	0.808 ± 0.035	0.928 ± 0.033	1.49 ± 0.31	3.47 ± 1.64	0.87 ± 0.19

SR indicates that the comparison is on the fine scale. The best results of each metric at native resolution are marked in bold.

**Figure 7:**
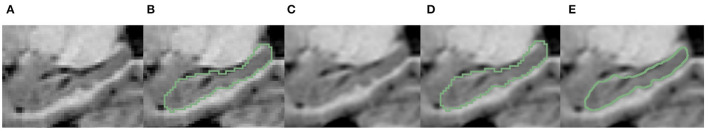
Visualization comparison of hippocampal segmentation results obtained at native image resolution and fine scale in EADC-ADNI dataset. **(A)** Shows the MR image of hippocampus at native resolution; **(B)** shows the segmentation results of hippocampus at native resolution in green contour line; **(C)** shows the MR image after interpolation at fine scale; **(D)** shows the native resolution hippocampus segmentation overlaid on the fine-scale MR image after interpolation; **(E)** shows the fine-scale hippocampus segmentation overlaid on the fine-scale MR image.

It is noteworthy that all the above segmentation results were obtained at the native image resolution. Although they all achieve quite high evaluation metrics, as can be seen in Figure 7C, at the sub-pixel level, the bumpy structure can be clearly seen. However, on the reconstructed surface, the staircase appearance does not indicate the correct local morphology. This is because all the methods above are based on training annotations. However, under the native resolution, even manual annotation can not correctly characterize the bumps. Because the function space limited by the native resolution determines the morphological characterization capability. Such a surface space limitation should be addressed, for the segmentation to correctly characterize the bumps/dentations. To solve this problem, we need the help of fine-scale segmentation to get the fine-scale annotations.

#### 3.1.3. Fine-scale segmentation for EADC-ADNI dataset

The fine-scale segmentation results for the EADC-ADNI dataset are shown in Figure 7E. It can be seen that the smooth curves accurately delineate the bumpy hippocampal structure. The last row in Table 3 shows the quantitative analysis results of the fine-scale segmentation results compared with the FSL+manually label of the EADC-ADNI dataset. As seen in that row, the value of the DSC metric increased by about 2% and the precision value also increased compared to Table 3. This may be due to the curvature regularization at a much higher resolution. While it successfully regulates the surface evolution from generating singularities, inevitably, it will shrink the total volume slightly and result in a more conservative segmentation.

It can also be seen from the quantitative results in the last row of Table 3, the fine-scale segmentation results have improvements in the DSC and Jaccard similarity coefficients compared to the native-resolution-based results. This is consistent with the visualization results shown in Figure 7, since these two metrics are volume-based evaluation metrics. Nevertheless, it still cannot reflect the changes in dentation segmentation significantly. Because fine-scale segmentation is reflected more on improving the accuracy on the boundary, rather than on the volumetric measurement. Therefore, surface-distance-based metrics, such as HD, can better demonstrate improvements in fine-scale segmentation. However, a full 3D delineation across the thousands of slices at a much higher resolution in a consistent manner is extremely tedious, if not impossible. To solve that dilemma, we use two-dimensional Hausdorff distance (2D HD) at certain characteristic slices for quantitative evaluation.

The characteristic sagittal slice that best reflects the bumpy features on the surface of the hippocampus is selected at fine-scale resolution. Then, the boundaries of hippocampal dentation are outlined manually. After that, for comparison, the fine-scale hippocampal annotation on the same slice was extracted. The Hausdorff distance between its boundary and the manually drawn contour was calculated. The results are shown in Table 3 (the “2D HD” column). As can be seen, the segmentation of dentation at fine scale is significantly improved reflected by this evaluation metric. This indicates that the fine morphology is better captured at such a fine-scale resolution than that at the native image resolution. It is also consistent with the visualization results shown in Figure 7, with more accurate dentation annotation at the fine scale.

### 3.2. Segmentation evaluation of IXI dataset

The ultimate goal of performing fine-scale segmentation is to extract the detailed hippocampal morphology, which can be used for cross-sectional and/or longitudinal comparisons among different groups of subjects. With such a goal in mind, while the EADC-ADNI dataset has manual annotation at the native image resolution, we have to deploy the algorithm to a much larger set for the morphometry. Unfortunately, such a dataset of 552 healthy subjects does not have a complete reference annotation.

In this section, the fine-scale segmentation is carried out and evaluated on such a much larger dataset.

#### 3.2.1. Segmentation comparison at native resolution

As mentioned above, since the IXI dataset does not provide annotations of the hippocampus, following (Liu et al., 2020), we obtained the hippocampus segmentation with the help of FSL software and manual correction as the reference ground truth in IXI dataset. Then, it is used to evaluate the segmentation performance of the proposed framework and other SOTA algorithms on the IXI dataset. The experiments described next are based on the randomly selected 150 sample subjects from the IXI dataset as the research objects.

**Figure 8:**
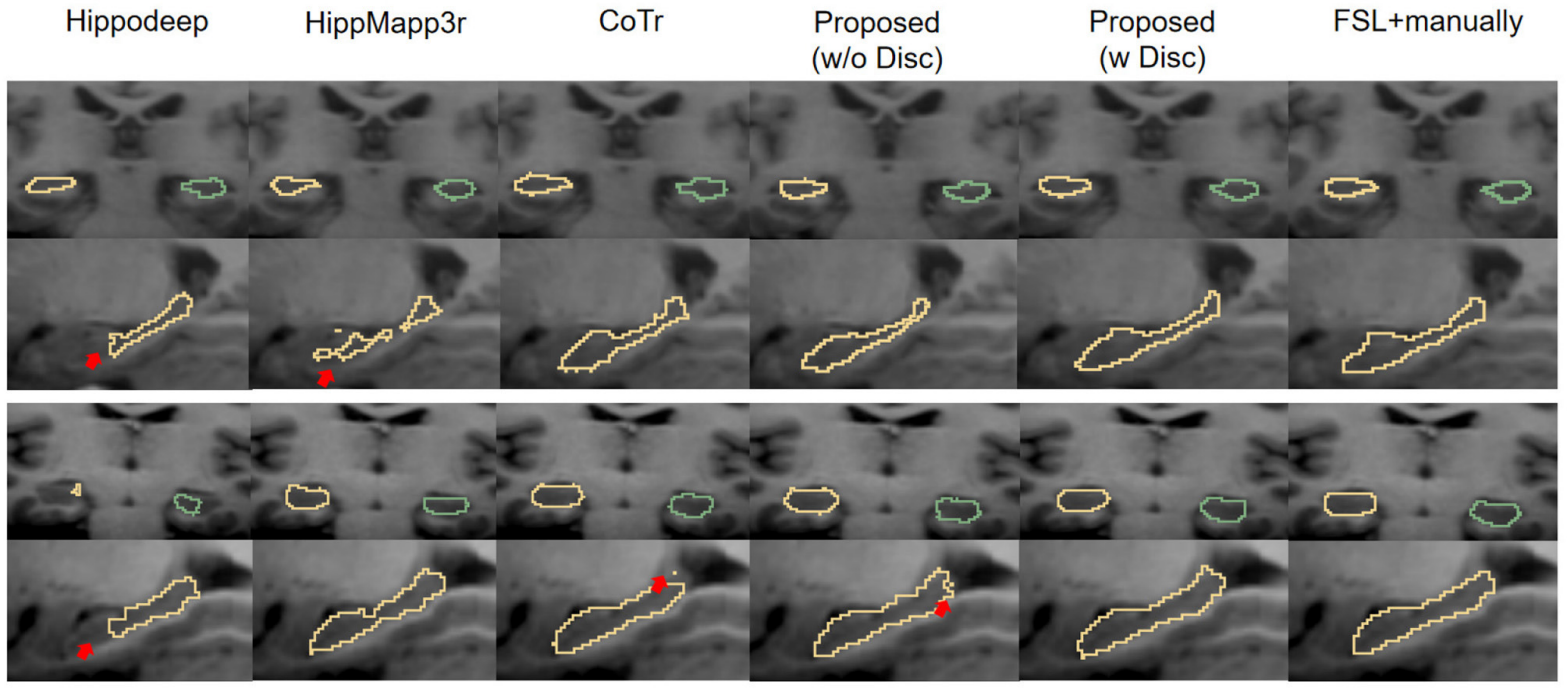
Visual contour and comparison of different segmentation methods on two example subjects from the IXI dataset. The first and second rows are from the first subject, and the third and fourth rows are from the second subject. Yellow and green outlines indicate left and right hippocampus segmentation. Manually traced results are displayed in the last column on the right. The red arrows indicate the over- or under-segmentation error.

Figure 8 shows the visual comparison of the segmentation results given by different segmentation models at the native image resolution. It shows that Hippodeep fails to capture the head of the hippocampus. Likewise, HippMapp3r does not perform well in the same example subject. The output of CoTr is incomplete but unlike the previous examples, it omits the caudal part of the hippocampus. Improved from CoTr, our proposed basic segmentation framework (without discriminator) is not constrained by the input size and can output more complete segmentation results.

The reason for the under-segmentation of the IXI dataset by these above methods may be that they were not directly trained by the annotations of the IXI dataset, and the distribution of hippocampal samples on the IXI dataset has not been seen before. In contrast, the proposed framework with discriminator can utilize the new images to adaptively improve the generalization ability of the model. Therefore, the proposed framework performs well in these samples, and its output is visually closer to the ground-truth annotations.

Table 4 shows the quantitative results using different methods on the 150 sample objects of IXI dataset. Combining the visualization results in Figure 8 and the quantitative analysis results in Table 4, we have the following findings.

**Table 4:** Quantitative comparison of segmentation results obtained by different segmentation methods with results from FSL+manual annotation on IXI dataset.

**Framework**	**Left_Right**	**DSC↑**	**Jaccard↑**	**Recall↑**	**Precision↑**	**HD95 (mm)↓**	**HD (mm)↓**	**2D HD (mm)↓**
Hippodeep (Thyreau et al., 2018)	Right	0.835 ± 0.070	0.721 ± 0.077	0.814 ± 0.076	0.861 ± 0.069	1.92 ± 1.64	4.47 ± 1.86	1.72 ± 1.62
	Left	0.835 ± 0.067	0.721 ± 0.073	0.806 ± 0.076	0.873 ± 0.065	1.89 ± 1.69	4.32 ± 1.97	1.75 ± 1.96
HippMapp3r (Goubran et al., 2020)	Right	0.812 ± 0.053	0.686 ± 0.062	0.735 ± 0.059	**0.911** **±** **0.071**	1.91 ± 0.65	4.34 ± 1.21	1.91 ± 0.54
	Left	0.807 ± 0.050	0.678 ± 0.056	0.720 ± 0.061	**0.922** **±** **0.065**	1.95 ± 0.51	4.16 ± 1.11	1.74 ± 0.59
CoTr (Xie et al., 2021)	Right	0.846 ± 0.017	0.734 ± 0.025	0.821 ± 0.028	0.876 ± 0.039	1.68 ± 0.32	4.08 ± 0.52	**1.67** **±** **0.95**
	Left	0.850 ± 0.019	0.740 ± 0.028	0.824 ± 0.027	0.880 ± 0.038	1.69 ± 0.30	4.02 ± 0.61	1.56 ± 0.87
Proposed (w/o Disc)	Right	0.850 ± 0.028	0.741 ± 0.04	0.806 ± 0.045	0.902 ± 0.039	1.69 ± 0.34	**3.86** **±** **0.62**	2.03 ± 1.41
	Left	0.854 ± 0.030	0.746 ± 0.043	0.806 ± 0.042	0.909 ± 0.040	1.73 ± 0.36	3.78 ± 0.66	1.93 ± 1.42
Proposed (w Disc)	Right	**0.865** **±** **0.020**	**0.763** **±** **0.030**	**0.841** **±** **0.036**	0.894 ± 0.039	**1.60** **±** **0.31**	3.98 ± 0.64	1.78 ± 1.22
	Left	**0.867** **±** **0.021**	**0.765** **±** **0.033**	**0.843** **±** **0.031**	0.894 ± 0.041	**1.63** **±** **0.33**	**3.70** **±** **0.70**	**1.52** **±** **1.35**
Proposed (SR)	Right	0.866 ± 0.022	0.765 ± 0.033	0.835 ± 0.039	0.903 ± 0.045	1.40 ± 0.29	3.17 ± 0.50	1.24 ± 0.96
	Left	0.865 ± 0.023	0.763 ± 0.035	0.837 ± 0.036	0.897 ± 0.04	1.50 ± 0.28	3.08 ± 0.55	1.25 ± 1.39

SR indicates that the comparison is on a fine scale. The best results of each metric at native resolution are marked in bold.

First, the Dice score of Hippodeep is higher than that of HippMapp3r. However, Hippodeep has certain unsatisfactory segmentation as shown in Figure 8, resulting in a larger standard deviation. Additionally, the precision of the segmentation of HippMapp3r is the highest among all methods. As can be seen in Figure 8, the output of HippMapp3r was more likely to be located within the hippocampal region. Therefore, the segmentation results of HippMapp3r have lower false positives and thus the highest precision. As for the evaluation results based on HD and 2D HD distance metrics, the proposed model achieves the best results on the left hippocampus than that on the right. Furthermore, compared with other frameworks, our proposed segmentation model achieves the best results on the other evaluation metrics. In particular, it is approximately 5% higher than the evaluation metric of HippMapp3r on DSC. To sum up, according to the quantitative and qualitative comparison results, the proposed framework's output is more satisfactory than hippocampal segmentation results on the IXI dataset.

Moreover, another advantage over state-of-the-art methods (Hippodeep and HippMapp3) is that only 30 subjects from another dataset (EADC-ADNI dataset) are used for training, while the testing set in this study involves a different cohort with 150 sample subjects. This shows the generalization capability of the proposed method.

#### 3.2.2. Evaluation of fine-scale segmentation

**Figure 9:**
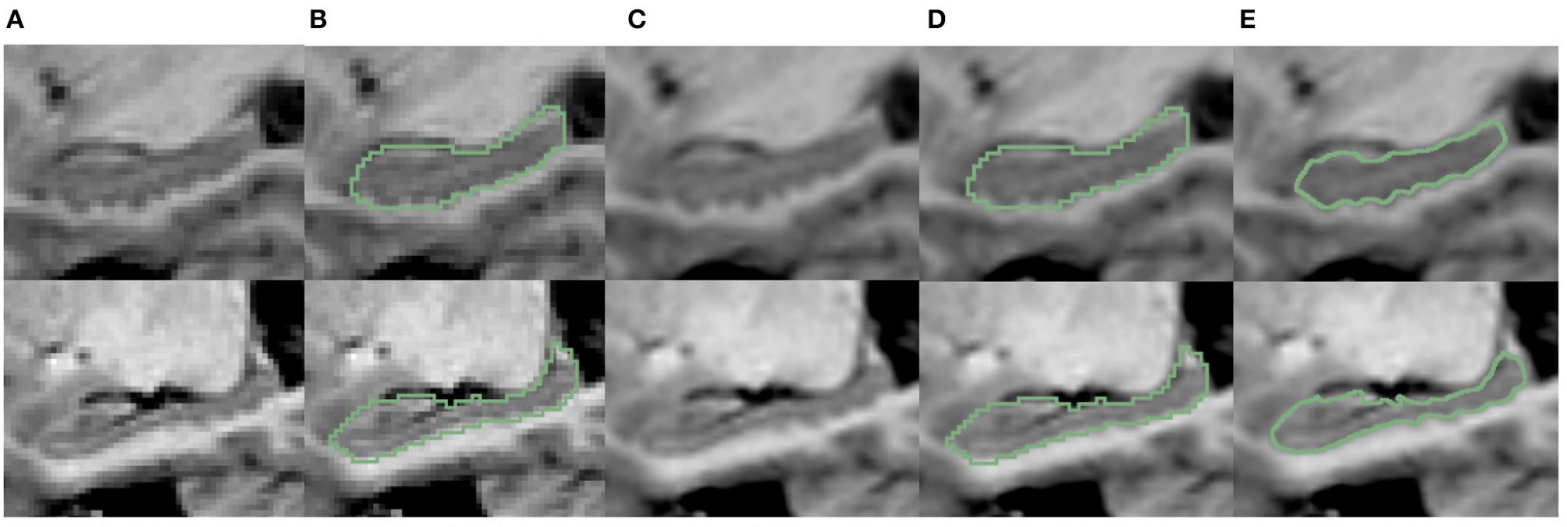
Visualization comparison of hippocampal segmentations at native image resolution vs. fine scale in IXI dataset. **(A)** The MR image of the hippocampus at native resolution. **(B)** The segmentation of hippocampus at native resolution; **(C)** shows the MR images after interpolation; **(D)** shows the native resolution hippocampus segmentation overlaid on the fine-scale MR image after interpolation; **(E)** shows the fine-scale hippocampus segmentation overlaid on the fine-scale MR image.

The above results are based on the native image resolution. To accurately analyze the change of dentation, we applied the fine-scale segmentation method to obtain the segmentation and compare them with manual annotation at native resolution and fine-scale, visually shown in Figure 9. Similar to Figures 7B, D, it can be seen from Figures 9B, D that the segmentation model can only output rough stepped edges at the native image resolution, but fails to capture the edges of the hippocampal dentation. Further, compared with the segmentation results in Figures 9B, D, the Figure 9E shows that the proposed method can better capture the dentation structure of the hippocampus, resulting in finer segmentation results.

The last row of Table 4 shows the quantitative analysis of the fine-scale segmentation results. Compared to the performance at native resolution, overlap-based metrics (i.e., DSC, Jaccrd, Recall, and Precision) did not show significant changes. But the distance-based metrics (i.e., HD95, and HD) decrease significantly. Among them, the HD metric can be lowered by up to 0.8 mm (about 20% for the right hippocampus). To measure the improvement of boundary fineness by fine-scale segmentation methods, we also focused on the results for 2D HD. There is also a 0.5 mm (about 28%) drop for the right hippocampus in the 2D HD metric for dentation.

Combining the overlap-based and distance-based metrics shows that the fine-scale segmentation algorithm does not have a great impact on the segmentation accuracy of the overlapped region of the hippocampus. Instead, the algorithm can change the edges of the segmented objects, thereby improving the accuracy of the dentate segmentation.

**Figure 10:**
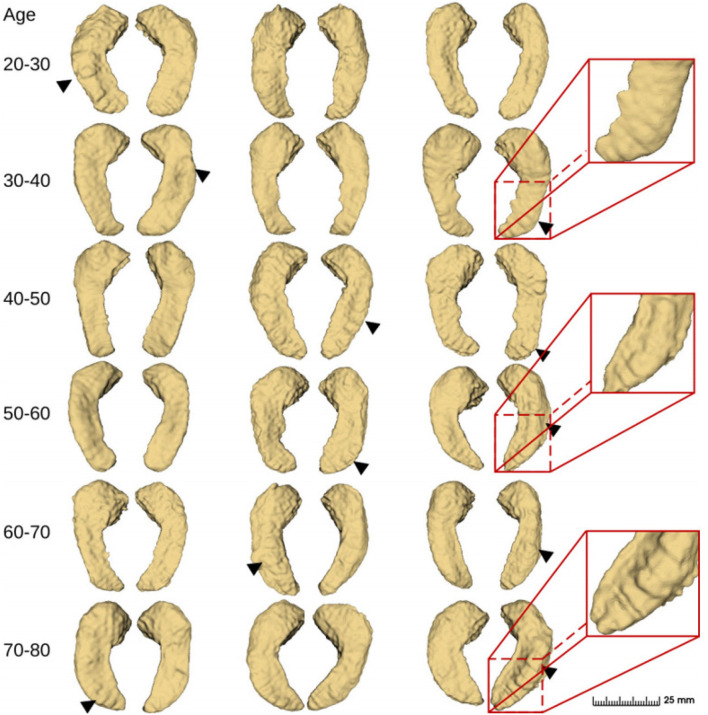
Fine-scale segmentation by the proposed method in different age groups from the IXI dataset (2018). Black triangles indicate hippocampal dentation.

Finally, we applied the proposed method and obtained fine segmentation results for all 552 case samples based on the IXI dataset. Some of the visual results are shown in Figure 10. The bumps on the inferior side of the hippocampus can be captured in the presented samples and they look different among different age groups. However, the “bumpiness” across different age groups can not be easily assessed by eye, and we need to use quantitative metrics to do so. This is subject to the topic of the next section.

Since the most important bumpy dentation information can be observed in the 2D slices, we segmented the volumetric data and validated the accuracy in 2D.

### 3.3. Validation with 7T MR images

**Figure 11:**
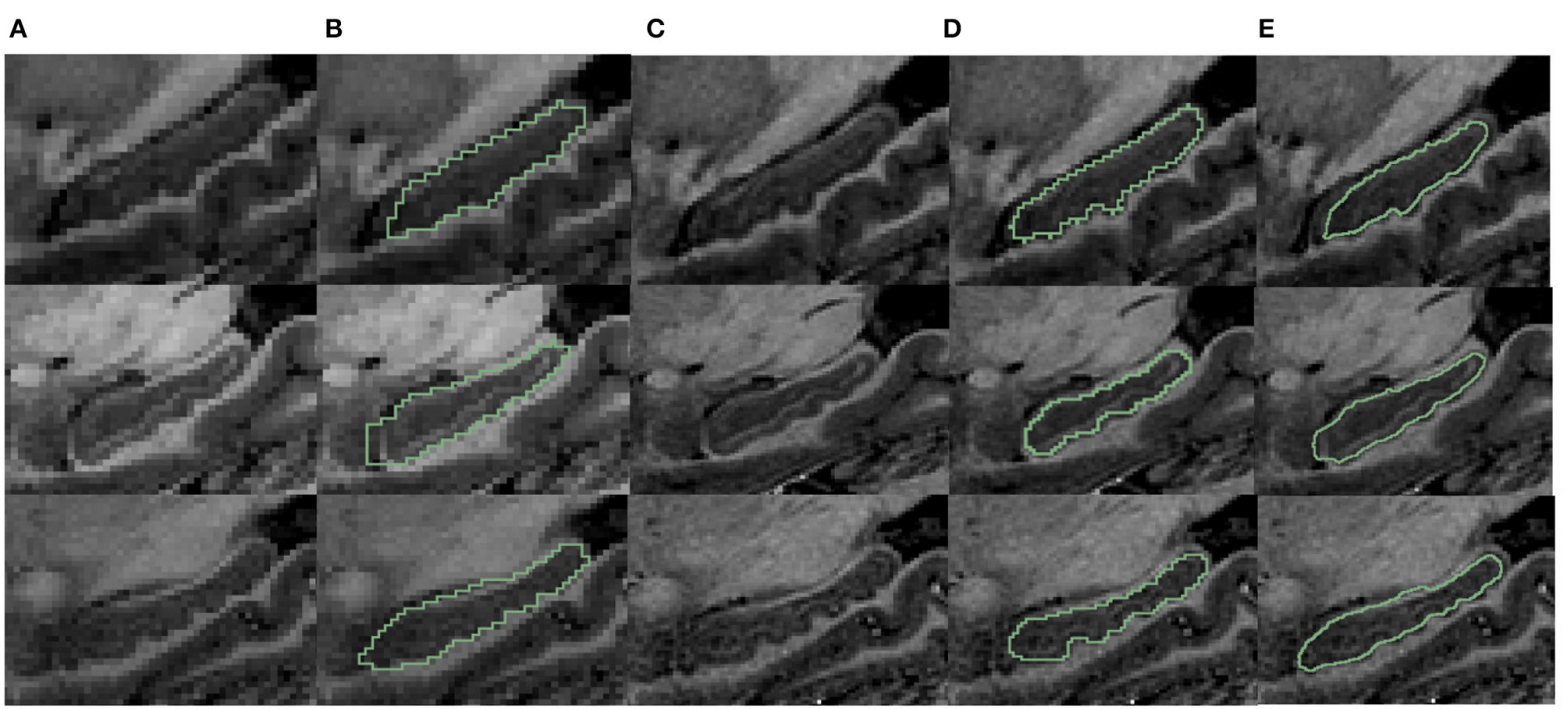
Visualization comparison of hippocampal segmentations in 7T MR scans. **(A)** The MR image of the hippocampus at resampled 1 mm resolution. **(B)** The obtained segmentation of the hippocampus at 1 mm resolution overlaid on **(A)**; **(C)** shows the MR images at native 0.641 mm resolution from 7T scans; **(D)** shows the manual annotation overlaid on the 7T MR images; **(E)** shows the fine-scale hippocampus segmentation with resolution 0.2 mm overlaid on the native 7T MR image with resolution of 0.641 mm.

To validate our segmentation accuracy on 1 mm/pixel MR scans against high-resolution 7T MR scans, we mimic lower resolution images using 7T MR scans, applied the proposed method, and validated the result against the manual contour of the inferior surface slices which shows the most prominent bumps at high resolution. For analysis, we selected three samples from the dataset provided by Alkemade et al. (2020) for testing. Since the most important bumpy dentation information can be observed in the 2D slices, we segmented the volumetric data and validated the accuracy in 2D. First, we down-sampled the 7T MR images with a resolution of 0.641 to 1 mm. We then applied the proposed segmentation framework to obtain fine-scale segmentations. The segmentation results were presented in Figure 11. Observing Figures 11B, C, it can be seen that the native segmentation results at 1 mm resolution in Figure 11B cannot accurately capture the boundaries of hippocampus dentation well, though it can be observed in Figure 11C, the original high-resolution slices. Conversely, the fine-scale segmentation results shown in Figure 11E, obtained with the proposed framework, exhibited a high degree of consistency with the manual annotation in Figure 11D. Quantitatively, the DSC obtained on the three slices were 0.892, 0.897, and 0.861, respectively, while the 95th percentile of the Hausdorff distance measured 0.641, 0.640, and 0.906 mm. These results demonstrate that our fine-scale segmentation approach yields accurate outcomes, which closely align with the true 7T segmentation results obtained from high resolution MR scans.

### 3.4. Shape analysis of the hippocampal dentation in fine scale

Sections 3.2 and 3.1 above validated the segmentation accuracy of the proposed framework. The ultimate goal of the present work is to quantitatively analyze the fine-scale dentation feature underneath the hippocampus using the methods in Section 2.2. First, we quantitatively validated the hippocampal dentation analysis method in Section 2.2 on some simulated shapes in Section 3.4.1. We then applied the validated methods on the real fine-scale segmentation results in Section 3.4.2 and identified the trends of hippocampal dentation through different age groups.

#### 3.4.1. Quantitative validation of hippocampal dentation analysis on simulated shapes

In this section, we quantitatively evaluate the hippocampal dentation analysis method used in Section 2.2 and show its accuracy in capturing the magnitude and frequency of dentation patterns.

Since there is no established ground truth for the measurements of the dentation patterns, it would be difficult to evaluate the accuracy if we directly apply the methods to the real anatomical structures. As a result, following the ideas in Gao et al. (2014), we generated a series of simulated shapes, with known varying dentation patterns in their magnitudes and frequencies. Such shapes are then used to evaluate the analysis method.

**Figure 12:**
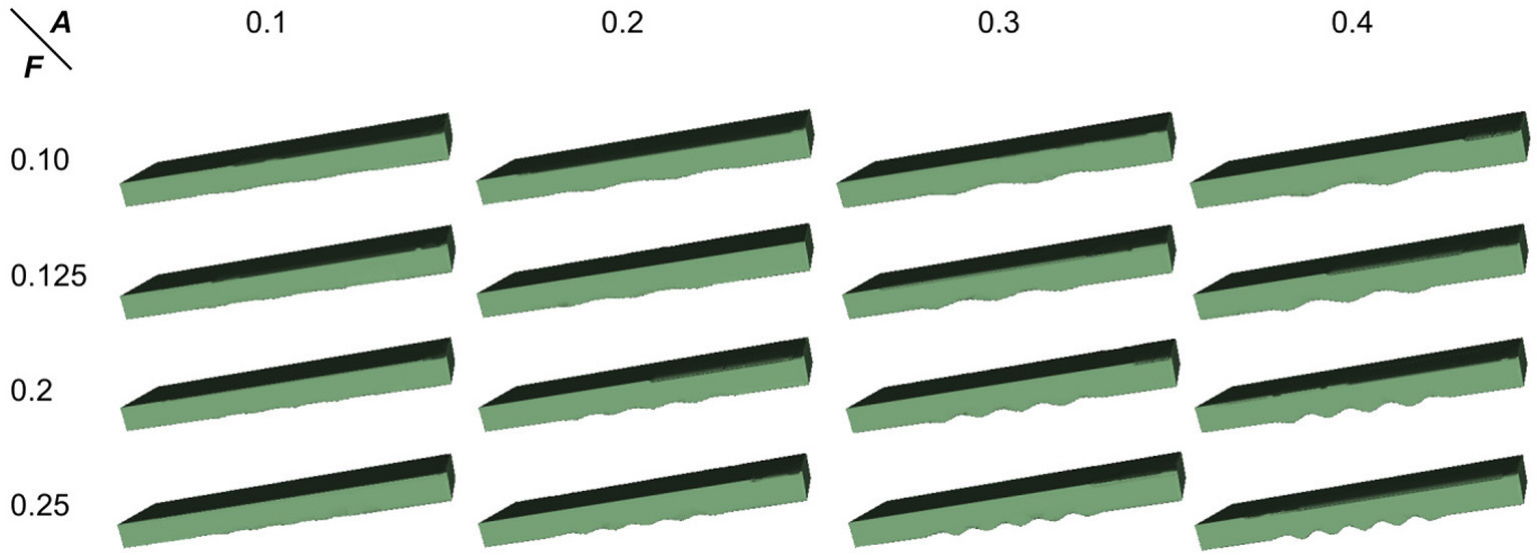
Simulated 3D hippocampal dentation. There are 16 combinations of dentation simulations with different amplitudes (***A***, ranging from 0.1 to 0.4 mm) and frequencies (***F***, ranging from 0.1 to 0.25 bump/mm).

**Figure 13:**
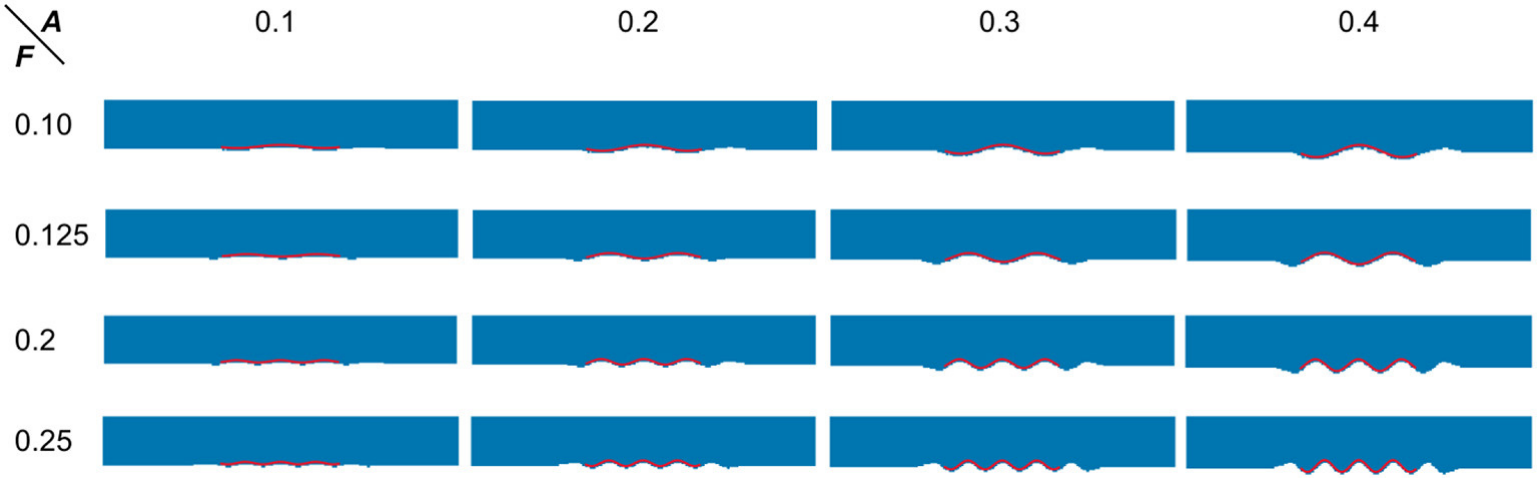
Visual examples of dimensionality reduction and curve fitting. Blue points represent the result of the 3D simulation model after dimensionality reduction. The red wavy curve represents the fitted curve.

To proceed, we simulated the dentations with dentation amplitudes and frequency ranges concerning (ten Hove and Poppenk, 2020) on a cuboid, as shown in Figure 12. Then, following the proposed shape analysis method, we extracted both the amplitudes and the frequencies. Corresponding to the simulation example in Figure 12, the visual examples of the results obtained from dimensionality reduction and curve fitting are shown in Figure 13. Based on the fitted curve, we computed the dentation frequencies and amplitude. Finally, the computed amplitudes and frequencies are compared with their ground truth. The evaluation results are shown in Table 5.

**Table 5:** Quantitative error analysis results between the fitting and the actual setting frequency (***F***) and amplitude (***A***).

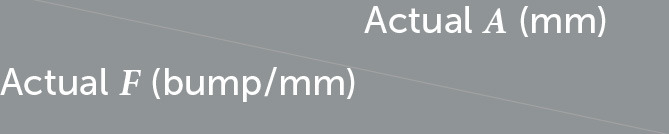	**0.1**	**0.2**	**0.3**	**0.4**	**MAE (*F*)**
					
0.1	0.082 (0.101)	0.193 (0.099)	0.297 (0.1)	0.401 (0.1)	0.001
0.125	0.082 (0.124)	0.19 (0.124)	0.299 (0.124)	0.398 (0.125)	0.001
0.2	0.082 (0.2)	0.191 (0.199)	0.295 (0.2)	0.394 (0.2)	0
0.25	0.08 (0.251)	0.189 (0.249)	0.294 (0.25)	0.396 (0.25)	0.001
MAE (***A***)	0.019	0.009	0.004	0.003	\

In parentheses are the fitted frequencies.

As can be seen from Table 5, the fitting error of the frequencies is below 1%. This is partially because the shapes are quite ideal. However, the error of the amplitude detection is larger, with a maximum error of about 0.019 mm. It can also be observed from Table 5 that the amplitudes are often time underestimated. However, when the amplitude is larger than 0.2 mm, the statistical error in Table 5 is greatly reduced to around 4.5%. As the amplitude increases to 0.4 mm, the fitting error is lower than 1%. Consistent with this, it can also be seen from Figure 13 that the fitted curve is almost the same as the actual curve.

The above quantitative analysis of errors shows that, based on dimensionality reduction and curve fitting, the proposed shape analysis method has relatively larger errors when the bumps are shallow. With the gradual increase of the bump amplitude, the error decreases to about 1%. After this validation of the simulated data was completed, we then applied the method to the real segmentation results.

#### 3.4.2. Quantitative analysis of hippocampal dentation —The hippocampus is the bumpiest in people in their 40s

**Figure 14:**
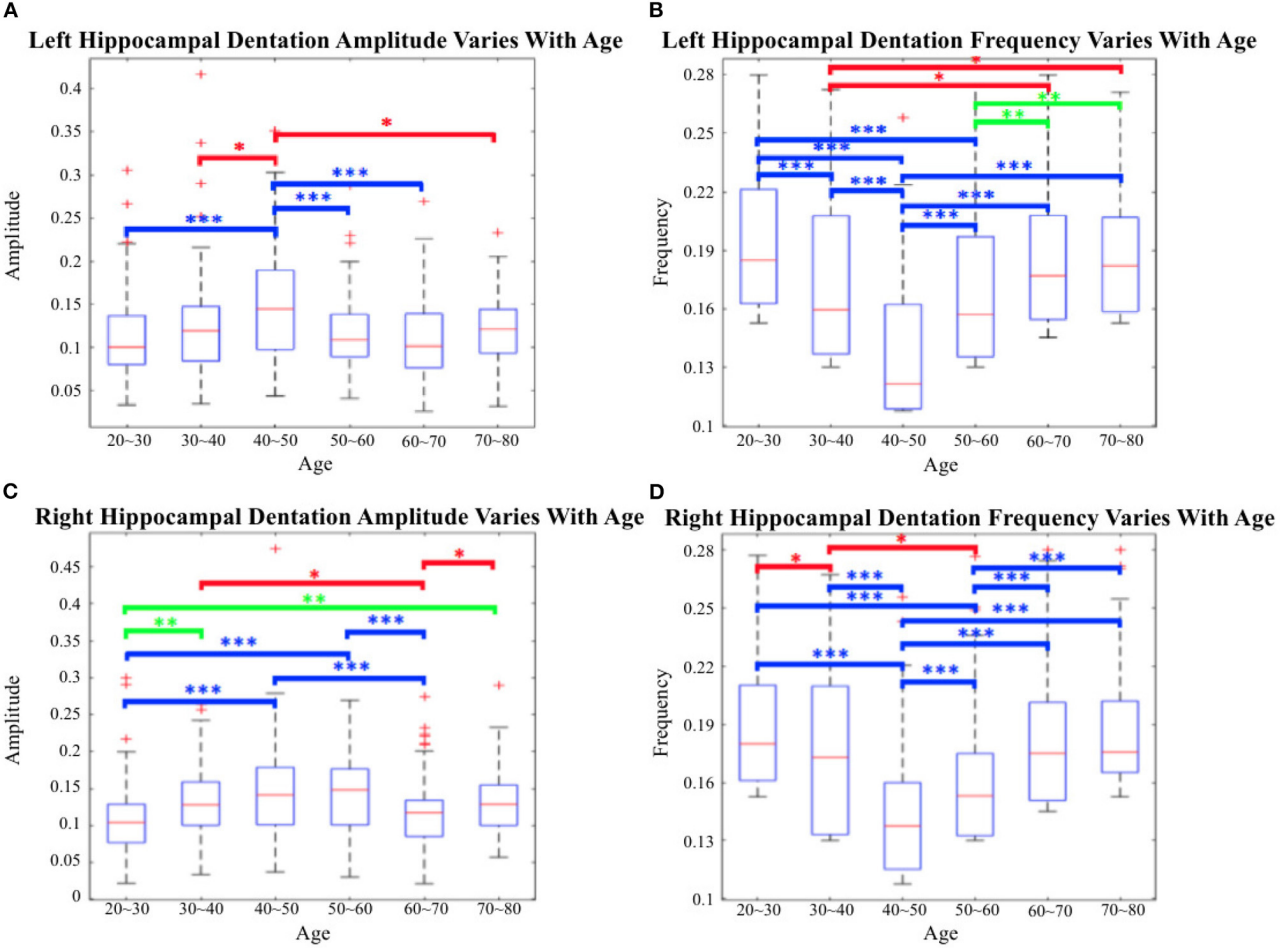
Cross-age group assessments. **(A, C)** Box-plots of left hippocampal dentation amplitudes and frequency variation with age; **(B, D)** Box-plots of right hippocampal dentation amplitudes and frequency variation with age. The bridges of different colors span between the two groups, indicating that their difference is statistically significant. *P < 0.05 (indicated in red); **P < 0.005 (indicated in green); ***P < 0.001 (indicated in blue).

Utilizing the proposed segmentation method, we captured the fine-scale dentations on the IXI dataset. Shape analysis was subsequently performed on the annotated data from the IXI dataset, consisting of 552 healthy subjects, using the method described in Section 2.2. Figure 14 shows the variation in hippocampal dentation amplitude and frequency sub-stratified by age. The higher the amplitude, the higher the hippocampus dentation. Higher frequencies indicate narrower dentation in the hippocampus.

As depicted in Figure 14, the dentations under the hippocampus are most pronounced in the age group of people between 40 and 50 years old. First, there were more variations of amplitude in the 40 to 50 age group, which ranged approximately from 0.09 to 0.2 on both sides in Figures 14A, C. On the other hand, the change in frequency trended in the opposite direction to the change in amplitude but still reached its lowest point at the age of 40 to 50, and ranged from 0.11 to 0.16.

Figure 14 shows inter-group statistical analysis and the differences by two sample independent *t*-tests. The temporally aligned blocks for six groups reveal distinct (*P* < 0.05) patterns in hippocampus dentation. The most notable differences between groups were the amplitude of left hippocampus dentation (group 40–50/others).

## 4. Discussion and conclusion

This work has presented a complete pipeline of fine-scale hippocampus segmentation and dentation analysis. Results indicated that this is an efficient method for accurate sub-millimeter hippocampus segmentation and dentation shape variation analysis in 3T MR images in different age groups.

The proposed method addressed the two main difficulties of obtaining fine-scale annotation of the hippocampus efficiently from clinically available image data instead of ultra-high field MR scans and exploring the relationship between hippocampal longitudinal dentation and age in normal and healthy groups.

For hippocampus segmentation, the proposed algorithm based on 3D deep neural networks improved the segmentation performance and efficiency, which fulfilled the need to obtain annotation of the hippocampus of a large cohort with 552 sample subjects. Only a small sample size of 30 volumes was used for model training and hippocampus segmentation tasks. To solve the problem of the difference in the distribution of training and testing samples, we improved from the CoTr model and utilized the domain adaptation method to improve the performance of validation and testing on the second dataset. For example, the segmentation performance of the tail in the hippocampus was improved. This deep learning based semantic segmentation method provided accurate initial segmentation for the subsequent fine-scale segmentation. Furthermore, to compare the change of hippocampal segmentation results at the native resolution and the fine scale, we applied distance-based evaluation metrics. The reduction of HD and 2D HD showed that, with the help of fine-scale segmentation algorithms for morphological analysis, segmentation results could better capture the outline of the whole hippocampus and its dentation.

To the best of our knowledge, this is the first quantitative investigation of fine-scale hippocampus morphometry across a wide range of age groups. This initial study reveals noticeable patterns of shape changes. Dentation of the hippocampus is present during the initial stages of life and continues to change as the individual grows. These changes are commensurate in relative extent with the temporal structural evolvement of the hippocampus within the first few decades up to the age of 50. By contrast, the dentational region undergoes a lower rate of change, leading to a relative degree of loss in the inferior regions of the hippocampus. Although the total change rate of dentational regions presents concavity or convexity for the corresponding quantitative parameters, the reverse is not true for people in older age groups: in these individuals, with severe tissue loss, dentation has a more irregular outline.

These findings are consistent with continuous variability across the full spectrum of neurogenesis, as is increasingly being verified from the molecular structure level (Alvarez-Buylla and Lim, 2004; Lim and Alvarez-Buylla, 2016). Another study, in Wu et al. (2013), has demonstrated that the structural brain network also peaks between 40 and 50 years of age.

Our findings support the idea that the temporal profiles of dentation in healthy subjects may be the consequence of neurogenesis at the specific site of brain regions. It has also been demonstrated that adults preserve neural stem cells, which produce new neurons within some restrained areas. These cell populations could be viewed as displaced and modified neuroepithelium, pockets of cells, and local signals that retain enough embryonic nature to maintain neurogenesis for life. These findings suggest a selective cortical variation that is consistent with the extent and dynamics of neurogenesis, with the most active growth happening during embryonic development, followed by continuous generation, decreasing slowly with age (Knoth et al., 2010; Sanai et al., 2011; Göritz and Frisén, 2012). However, regions of neurogenesis exhibit pathological distinctions between healthy subjects and some neurodegenerative disease patients, and these distinctions are evident throughout the course of a disease. For example, in Huntington's disease, it has been found that postnatally generated neurons are absent in the advanced stages of disease (Zuccato et al., 2010; Walker et al., 2011). Accordingly, serious consideration should be given to which factors might result in distinctions of neurogenesis activity, and whether there is any associated phenotype, just as Huntington's disease subjects seemed to reveal a more pronounced rate of atrophy within specific regions of interest. Similarly, longitudinal model-based estimation of variations and distinct phenotypic variability of dentation compared to healthy subjects could not be neglected during some neurodegenerative conditions.

A key advantage of this work is that it develops methods for quantifying the continuous phenotypic variability of dentation, which ranges from completely absent to pronounced among healthy adults. The proposed method extracts prominent change patterns from 3D volume data, which are critical for subsequent evaluation and to provide an effective feature expression. Compared to previous cross-sectional studies (Beattie et al., 2017), our work dispenses with a burdensome and subjective visual rating process. The non-linear fitting model provides two parameters—amplitude and frequency—to permit quantitative analysis of variation. However, the framework can only integrate dentation contour to a sinusoidal locus where the modeled average rate of change of mass data can support the model-based estimation.

The amplitudes and frequencies we measured are smaller than those found in ten Hove and Poppenk (2020). There may be several reasons for this: first, the IXI dataset we used are vanilla T1-MPRAGE sequence, which are not designed to highlight the hippocampal dentations. Second, in Section 2.2, we used a linear projection to map the 3D shape to 2D curves, which were later fitted with a sinusoidal function. In this process, the direction of projection may not be perfectly aligned with the ridge of the dentation due to its non-planner/non-linear nature. Furthermore, the inferior surface of the hippocampus is not a flat plane. The combination of these factors could decrease the amplitude of dentation in the 2D view and subsequent sinusoidal fitting. Further research investigating better bump extraction and parameterization approaches, such as the principal curve analysis and/or machine learning based approaches, is ongoing.

As the first systematic temporal study of hippocampal fine-scale dentation that includes analyses of 3T clinical data and comprehensive neuroanatomical measures, a few limitations to the present work have to be noted. Even though we validated that the dentations found in the proposed method are not interpolation artifacts, as seen in Chang et al. (2018), it is preferable to obtain paired 3T and 7T datasets for further validation of the dentation delineation. Moreover, the imaging data in this study were acquired from public databases. To enhance the robustness and generalization of the estimation model, promoting more studies spanning different databases from different sites and large-scale analysis to integrate these data is required. For instance, after the axis extraction, the non-linear fitting is susceptible to the local minimum. Even though the simulated annealing can ameliorate this situation to some extent, further improvements in dentation feature extraction need to be undertaken in future research.

In addition to the healthy subjects studied here, future directions of this research should also explore the potential diagnostic and prognostic utility of patterns of dentation in disease states, as well as serving as an outcome measure for interventions, such as epilepsy, Alzheimer's disease, and schizophrenia.

## Data availability statement

The original contributions presented in the study are included in the article/supplementary material, further inquiries can be directed to the corresponding author.

## Ethics statement

Ethical review and approval was not required for the study on human participants in accordance with the local legislation and institutional requirements. The patients/participants provided their written informed consent to participate in this study.

## Author contributions

QY and SC: methodology, software, and writing the manuscript. GC, RC, CH, and TR: writing the manuscript. XY and NZ: methodology. YG: conceptualization of this study, methodology, and writing the manuscript. All authors contributed to the article and approved the submitted version.
